# Modulation of tau tubulin kinases (TTBK1 and TTBK2) impacts ciliogenesis

**DOI:** 10.1038/s41598-023-32854-4

**Published:** 2023-04-14

**Authors:** Frances M. Bashore, Ariana B. Marquez, Apirat Chaikuad, Stefanie Howell, Andrea S. Dunn, Alvaro A. Beltran, Jeffery L. Smith, David H. Drewry, Adriana S. Beltran, Alison D. Axtman

**Affiliations:** 1 Structural Genomics Consortium, UNC Eshelman School of Pharmacy, University of North Carolina at Chapel Hill, Chapel Hill, NC 27599 USA; 2 Human Pluripotent Cell Core, University of North Carolina at Chapel Hill, Chapel Hill, NC 27599 USA; 3 Institute of Pharmaceutical Chemistry, Goethe University Frankfurt, Max-von-Laue-Str. 9, 60438 Frankfurt, Germany; 4 Structural Genomics Consortium, Buchmann Institute for Life Sciences, Goethe University Frankfurt, Max-von-Laue-Strabe 15, 60438 Frankfurt, Germany; 5 Department of Computer Science, University of North Carolina at Chapel Hill, Chapel Hill, NC 27599 USA; 6 Neuroscience Center, University of North Carolina at Chapel Hill, Chapel Hill, NC 27599 USA; 7 UNC Lineberger Comprehensive Cancer Center, School of Medicine, University of North Carolina at Chapel Hill, Chapel Hill, NC 27599 USA; 8 Department of Genetics, University of North Carolina at Chapel Hill, Chapel Hill, NC 27599 USA

**Keywords:** Chemical tools, Small molecules, Induced pluripotent stem cells, Screening, Structure-based drug design

## Abstract

Tau tubulin kinase 1 and 2 (TTBK1/2) are highly homologous kinases that are expressed and mediate disease-relevant pathways predominantly in the brain. Distinct roles for TTBK1 and TTBK2 have been delineated. While efforts have been devoted to characterizing the impact of TTBK1 inhibition in diseases like Alzheimer’s disease and amyotrophic lateral sclerosis, TTBK2 inhibition has been less explored. TTBK2 serves a critical function during cilia assembly. Given the biological importance of these kinases, we designed a targeted library from which we identified several chemical tools that engage TTBK1 and TTBK2 in cells and inhibit their downstream signaling. Indolyl pyrimidinamine **10** significantly reduced the expression of primary cilia on the surface of human induced pluripotent stem cells (iPSCs). Furthermore, analog **10** phenocopies TTBK2 knockout in iPSCs, confirming a role for TTBK2 in ciliogenesis.

## Introduction

Tau tubulin kinase 1 and 2 (TTBK1/2) are understudied serine/threonine/tyrosine kinases that belong to the casein kinase 1 superfamily. Their kinase domains have 88% identity, 96% similarity, and the same catalytic residues: lysine 63 and aspartic acid 164 (TTBK1) and lysine 50 and aspartic acid 141 (TTBK2)^1^. The non-catalytic domains of these enzymes are distinct from one another^2^. Phosphorylation of several characterized substrates and distinct physiological roles for TTBK1/2 have been described in key processes such as mitosis, ciliogenesis, microtubule dynamics, glucose and GABA transport, neurotransmission, and neuroinflammation^1–3^.

TTBK1 expression is localized to the brain, especially in the cytoplasm of cortical, hippocampal, and entorhinal cortex neurons, and it is overexpressed in neurodegenerative diseases^1,4,5^. Although TTBK2 is ubiquitously expressed in human tissues, higher expression has been reported in the cerebellar Purkinje cells, granular cell layer, hippocampus, midbrain, and substantia nigra^6^. Consistent with this expression, TTBK1/2 have described pathological roles specific to the central nervous system and brain, such as in Alzheimer’s disease (AD), frontotemporal dementia (FTD), amyotrophic lateral sclerosis (ALS), and a spinocerebellar ataxia type 11 (SCA11)^7^. Distinct neuronal interactors and phosphorylation substrates for TTBK1/2 have been identified and can be probed to understand how targeting these kinases impacts specific, disease-relevant pathways^8^.

As their names imply, TTBK1/2 are reported to phosphorylate tubulin and tau at multiple serine residues. These same epitopes are also hyperphosphorylated in neurofibrillary tangles (NFT), a key pathological hallmark in several neurodegenerative diseases, especially AD^4^. TTBK1 expression induces neurite and axonal degeneration in early AD pathology, it is upregulated in the frontal cortex of AD patients, and single nucleotide polymorphisms (SNPs) are associated with late-onset AD^2,5,9,10^. Abundant expression of TTBK1/2 in the brain coupled with their role of phosphorylating tau and leading to NFT supports that TTBK1/2 inhibitors may be therapeutically beneficial in AD^10,11^.

With respect to ALS and FTD, TTBK1/2 phosphorylate TAR DNA binding protein of 43 kDa (TDP-43) in vitro and in vivo^12^. Phosphorylation causes TDP-43 precipitation and aggregation in the cytoplasm of neurons, contributing to toxicity and eventual neuronal death^1^. TTBK1/2 co-localize with TDP-43 inclusions in the frontal cortex and spinal cord of FTD and ALS patients, respectively^1,7,12^. Reduction of TTBK1 mRNA decreased levels of phosphorylated TDP-43 in a pathological model, suggesting that TTBK1/2 inhibitors could provide relief for FTD/ALS patients.

A pathogenic variant in the *TTBK2* gene that results in premature truncations of its encoded protein causes a rare neurological disorder called SCA11, characterized by atrophy of cerebellar Purkinje neurons^1,6,13^. Inducible TTBK1 transgenic mice provide a model of cerebellar neurodegeneration with phenotypes reminiscent of spinocerebellar ataxia^7^. SCA11 alleles of TTBK2 are reported to interfere with ciliogenesis and cilium stability^13^. Accordingly, several centriolar and distal end proteins are TTBK2 substrates, TTBK2 recruitment enables requisite steps of cilia assembly, and TTBK2 is essential for regulating the growth of axonemal microtubules during ciliogenesis^14–17^. TTBK2 loss permits basal body docking to the plasma membrane while impeding transition zone formation and ciliary shaft elongation^18^. Emerging studies suggest that abnormalities in the length and frequency of primary cilia could be a key regulator in several brain diseases^19^.

Our program targeting TTBK1/2 was initiated based upon two complementary motivations. First, we are interested in the generation and use of kinase inhibitors as tools to increase understanding of signaling pathways that drive neurodegenerative disease. TTBK1/2 have described functions in the brain and have become sought after targets for neurodegenerative disease therapies. Second, we seek to identify high-quality tool molecules for understudied kinases. TTBK1/2 were identified as “dark” kinases by the NIH Illuminating the Druggable Genome (IDG) program. The IDG program is accelerating characterization of the proteome via stimulation of research around those proteins that are most poorly studied, referred to as “dark”^20^. We are supported by the IDG program to illuminate the dark kinome and are doing so via concurrent development of high-quality chemical tools and novel cell-based kinase assays.

**Figure 1:**
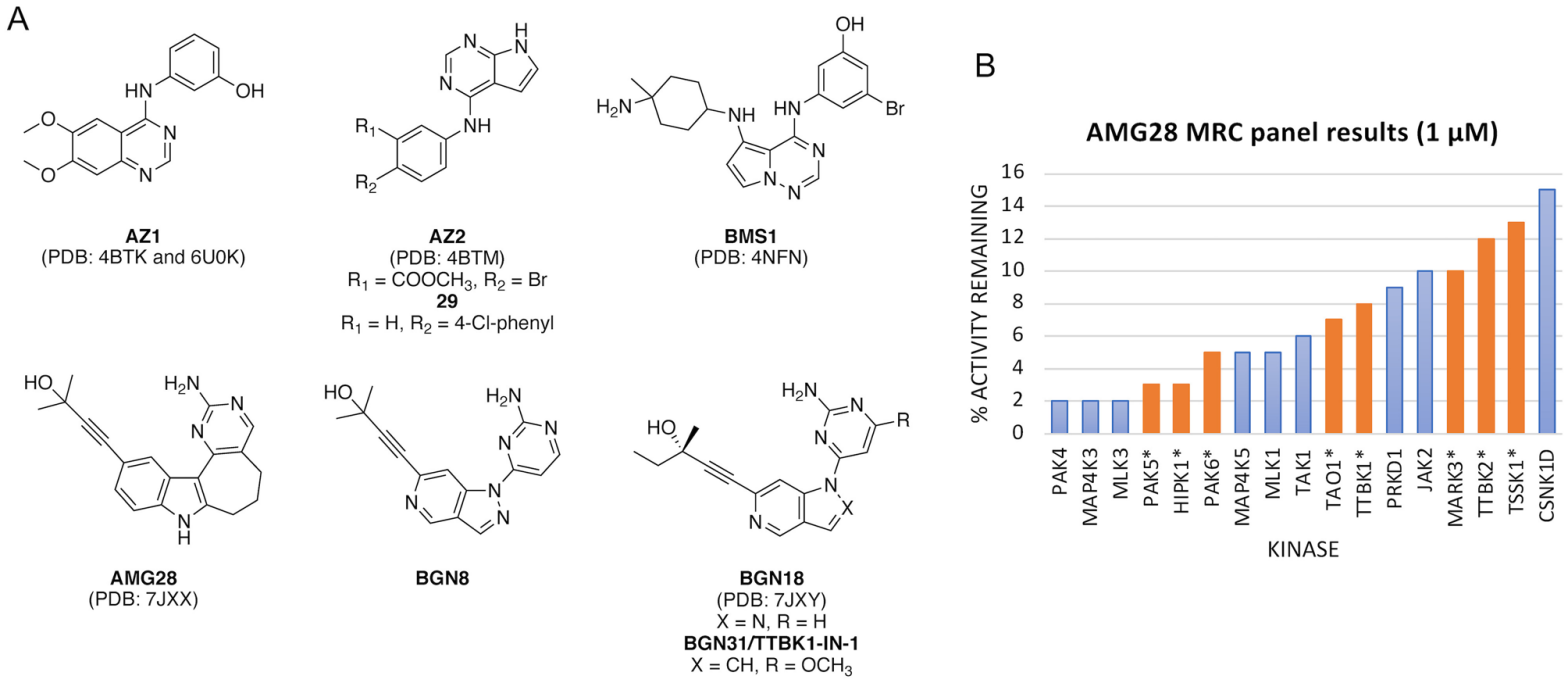
Structures and data for published TTBK inhibitors. (**A**) Structures of published TTBK1/2 inhibitors and PDB codes for corresponding structures. (**B**) AMG28 MRC panel screening results for kinases with < 15% activity remaining. IDG kinases indicated using asterisks and orange bars.

Interest in the development of TTBK1/2 inhibitors has recently increased. Co-crystal structures with TTBK1 have been deposited for inhibitors discovered by AstraZeneca, Bristol-Myers Squibb, and Biogen (AZ1, AZ2, BMS1, and BGN18, Fig. 1A)^21^. Biogen also solved the only co-crystal structure of TTBK2 bound to an inhibitor (AZ1, Fig. 1A)^22^. A recent paper described optimization of the AZ2 scaffold to yield compound 29 (Fig. 1A), which demonstrated good brain penetration and reduced TDP-43 phosphorylation in vivo^23^. A Biogen-led effort to deliver a potent and selective TTBK inhibitor with suitable CNS penetration for in vivo pharmacological studies resulted in BGN8, BGN18, and BGN31 (Fig. 1A). These inhibitors were developed to test hypotheses related to modulation of tau phosphorylation in hypothermia and developmental animal models^24^. BGN31 significantly lowered tau phosphorylation at multiple disease-relevant sites in vivo^11^. Of the inhibitors in Fig. 1A, extensive selectivity profiling data has only been reported for BGN18 and BGN31^24^.

Commercially available assays for TTBK1/2 are limited. A 2017 survey of biochemical kinase assays offered by the largest vendors identified that only half had available TTBK1/2 assays^25^. These kinases are not included in the largest kinome-wide profiling panel (Eurofins DiscoverX *scan*MAX), and thus the activity of broadly profiled compounds on TTBK1/2 could be overlooked. One panel that does include TTBK1/2 is that offered by the International Centre for Kinase profiling within the MRC Protein Phosphorylation Unit at the University of Dundee^26^. However, the translation of activity in these biochemical assays to a cellular context is unknown, and there is a need for target-specific cell-based assays to support medicinal chemistry optimization efforts aimed at delivering cell-active inhibitors.

The high homology of the kinase domains of TTBK1/2 makes it difficult to achieve selectivity between the two kinases as exemplified by the compounds in Fig. 1A, which display nearly equal potency for both enzymes. This selectivity complication aside, the most advanced inhibitors, developed by Biogen, have been used to explore biology mediated by TTBK1^11,24^. Our efforts have been dedicated to exploring a process ascribed to TTBK2: ciliogenesis. We have developed high-quality, cell-active, small molecule inhibitors of TTBK1/2 and illustrated their impact on ciliogenesis.

## Results

### Design and synthesis of indolyl pyrimidinamine inhibitors of TTBK1/2

**Figure 2:**
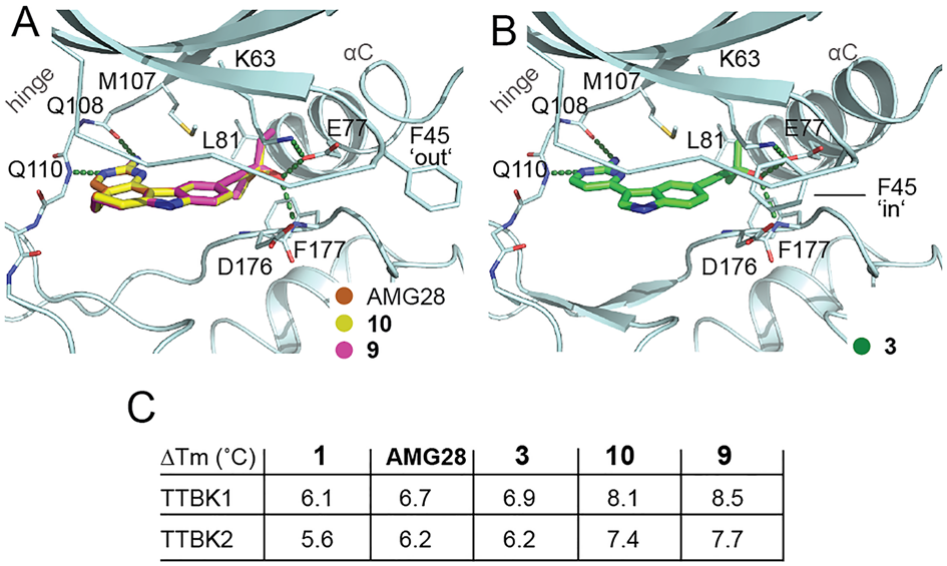
X-ray crystallographic structures of human TTBK1 in complex with several analogs and corresponding thermal shift data for TTBK1/2. (**A**) AMG28 (PDB code: 7ZHN) is shown in brown, **10** (PDB code: 7ZHQ) in yellow, and **9** (PDB code: 7ZHP) in pink stick representation, respectively. Image created using Schrödinger PyMOL 2.5. (**B**) **3** (PDB code: 7ZHO) is shown in pink. The hinge region and αC helix are labeled in each panel and hydrogen bonds are included as black dashed lines. Image created using Schrödinger PyMOL 2.5. (**C**) Table of thermal shift (ΔTm) data for TTBK inhibitors.

Our lead (AMG28, Fig. 1A) emerged from examination of the off-target activity of a published Amgen NF-κB inducing kinase (NIK) inhibitor^27^. The potency of AMG28 was reported in the MRC Kinase Profiling Inhibitor Database as 8% activity remaining for TTBK1 and 12% activity remaining for TTBK2 when screened at 1 μM (Fig. 1B)^26^. Biogen evaluated AMG28 in a biochemical assay (TTBK1 IC_50_ = 199 nM) and a cellular assay that measures inhibition of tau phosphorylation at Ser422 (IC_50_ = 1.85 μM). Like us (Fig. 2A), this group solved a co-crystal structure of AMG28 with the human TTBK1 kinase domain^24^.

Our first analogs were dedicated to understanding the role of the saturated ring system in AMG28 on TTBK1/2 activity. To investigate whether smaller or larger rings change the activity of this scaffold, we made the corresponding analogs that bear a six- and eight-membered ring, **1** and **2**, respectively. In addition, we removed the ring to make corresponding analog** 3**. These analogs maintained the substitution of AMG28 and only varied in the ring size or absence of a ring. We simultaneously explored changes to the substituent on the alkyne while retaining the seven-membered ring core scaffold (**4**–**11**). The free alcohol was unmodified in almost all analogs, with the exception of **11** in which the alcohol was capped as a methoxy group. Other analogs probe whether changing the groups attached to the carbon center bearing the alcohol to reduce the steric complexity (**4** and **5**) or increase their length from methyl to ethyl (**9** and **10**) is beneficial. Finally, the remaining three analogs (**6**–**8**) projected the alcohol further into the pocket by adding another methylene unit. These analogs vary in steric bulk around the alcohol to explore the tolerance of this deeper part of the pocket to these structural changes.

### Enzymatic kinase inhibition screening indicates TTBK1/2 inhibition

We screened our library in a 17-member enzymatic kinase assay panel (Table S1). Included kinases were selected based on the MRC panel screening results for AMG28 with a bias toward the inclusion of understudied IDG kinases, a designation annotated in Table S1 and in Fig. 1B for 14 of the 17 kinases^26^. In addition to TTBK1/2, PAK3–6, TAOK1–3, TSSK1–4, and MARK3–4 were included based on potent inhibition of kinase(s) in the family by AMG28 when profiled at 1 μM in the MRC panel (Fig. 1B). Like TTBK1/2, PAK, MARK, and TAOK family kinases have implications in neurodegenerative disease signaling^28^. Addition of HIPK1 and MAP4K5 completed our panel.

We profiled all 12 compounds (**1**–**11** and AMG28) at 1 μM against this kinase panel at the K_m_ value for ATP for each kinase. Table S1 contains these screening results as percent of control (PoC) for each analog for each kinase, with lower values indicative of greater inhibition. PoC data generated for each compound for TTBK1/2 is also included in Table 1. The column labeled “# < 10 PoC in enzyme panel” in Table 1 reports the number of kinases in this 17-member panel inhibited > 90% by each analog.

**Table 1:** Kinase panel profiling of indolyl pyrimidinamine library.


Compound	Scaffold	n	R	TTBK1 PoC/enzymatic IC_50_ (nM)	TTBK2 PoC/enzymatic IC_50_ (nM)	# kinases < 10 PoC in enzyme panel	S_10_(1 μM)^[a]^	# *scan*MAX kinases with PoC < 10^[b]^
**1**	A	1		78/3140	43/1210	2	0.05	20
**AMG28**	A	2		50/805	20/988	7	0.052	21
**2**	A	3		92/NT^[c]^	69/NT	1	NT	NT
**3**	B	N/A^[d]^		43/816	24/384	3	0.084	34
**4**	A	2		78/NT	64/NT	1	NT	NT
**5**	A	2		68/NT	72/NT	6	NT	NT
**6**	A	2		91/NT	76/NT	3	NT	NT
**7**	A	2		86/NT	104/NT	1	NT	NT
**8**	A	2		85/NT	103/NT	1	NT	NT
**9**	A	2		41/384	17/175	2	0.017	7
**10**	A	2		37/579	18/258	3	0.055	22
**11**	A	2		117/NT	110/NT	1	NT	NT

^a^S_10_(1 μM): percentage of screened kinases with percent of control (PoC) values < 10 at 1 μM. ^b^Number of kinases with PoC values < 10 at 1 μM. ^c^NT: not tested. ^d^N/A: not applicable.

This single-concentration data provided an initial indication of potency for TTBK1/2 as well as selectivity versus off-targets of parent molecule AMG28. We identified analogs of AMG28 that inhibited fewer kinases within this targeted enzymatic panel. For TTBK1/2 actives, defined as those demonstrating ≤ 50 PoC for TTBK1 and/or TTBK2, we observed a consistent trend that, like parent AMG28, all analogs were more potent inhibitors of TTBK2 than TTBK1. Analogs that maintained the propargylic alcohol were most tolerant of the seven-membered (AMG28) or no ring (**3**) when compared to analogs bearing six- and eight-membered rings (**1** and **2**, respectively). Capping the free hydroxy of AMG28 with a methyl group (**11**) was not favored. Analogs of AMG28 that removed a methyl group from the center bearing the alcohol (**4** and **5**) did not result in improvements in activity. In contrast, increased steric bulk around that carbon center by replacing one or more methyl group with an ethyl group (**9** and **10**) resulted in more potent TTBK1/2 inhibitors. Potency loss for the remaining three analogs (**6**–**8**) supported that the pocket is not tolerant of additional methylene groups on the alkynyl terminus. Seven-membered analogs confirmed that the TTBK1/2 binding pockets are sensitive to subtle changes in substitution around the hydroxy center and do not accommodate deeper projection of this group into the back pocket (Fig. 2).

### Kinome-wide selectivity profiling reveals that TTBK leads bind with high affinity to few kinases

Encouraged by the promising inhibition profiles in the 17-membered kinase panel, AMG28 and the four TTBK actives (**1**, **3**, **9**, and **10**) were next analyzed for kinome-wide selectivity at 1 μM via the DiscoverX *scan*MAX platform. This panel includes 403 wild-type (WT) human kinases but not TTBK1/2. The kinase for which AMG28 was originally designed, NIK, is among the 403 WT kinases^27^. Substantial overlap between the MRC and DiscoverX *scan*MAX kinase panels allows the comparison of results for the same kinases in two different assay formats (enzymatic versus binding, respectively) at the same concentration. Selectivity results are included in two columns within Table 1. The selectivity score (S_10_(1 μM)) was calculated for each analog and is equal to the percentage of 403 WT human kinases that exhibit a PoC < 10 at 1 μM. The rightmost column converts this selectivity score into the number of kinases with a PoC < 10 in the *scan*MAX panel. The WT kinases with a PoC < 10 for the five TTBK actives (AMG28, **1**, **3**, **9**, and **10**) are included in Fig. S1.

This more comprehensive selectivity analysis revealed interesting selectivity trends. The ring-opened analog **3** demonstrated the poorest kinome-wide selectivity. The six- and seven-membered ring containing analogs (**1** and AMG28, respectively) demonstrated improved kinome-wide selectivity compared to **3**. As the targeted enzymatic assay screening results suggested, each replacement of a methyl group with an ethyl group in **9** and **10** resulted in increased kinome-wide selectivity. Analog **10** bears a very similar substituted alkyne to the most selective TTBK inhibitors published by Biogen (BGN18 and BGN31, Fig. 1A), only differing by their choice to isolate a single enantiomer (S) versus our racemate (**10**).

### Orthogonal enzymatic studies validate binding and inhibition of TTBK1/2 and other kinases

We next evaluated compounds in TTBK1/2 enzyme assays in dose–response format. Except for AMG28, which was equipotent on both kinases, all analogs were more potent inhibitors of TTBK2 than TTBK1. The six-membered ring analog (**1**) was weakest in the TTBK1/2 enzymatic assays (IC_50_ values > 1 μM). While the ring-opened analog (**3**) and AMG28 were equipotent on TTBK1 (IC_50_ values 805–816 nM), **3** exhibited improved TTBK2 potency (**3**: IC_50_ = 384 nM; AMG28: IC_50_ = 988 nM, Table 1). Biogen scientists also prepared **3** and reported a five-fold or greater loss in potency relative to AMG28 in their biochemical and cell-based assays^24^. Analogs **9** and **10** are the most potent inhibitors of TTBK1/2 bearing a seven-membered ring (IC_50_ < 600 nM for TTBK1 and < 300 nM for TTBK2, Table 1). This enzymatic result corroborates the single-concentration data (Table S1), which predicted these two analogs to be the most potent inhibitors. It also aligns with the Biogen series, where they found the addition of an ethyl group provides a boost in potency due to displacement of a water molecule from the hydrophobic back pocket and van der Waals interactions between the ethyl group and lipophilic residues L81 and F177^24^. When considering the enzymatic data and kinome-wide selectivity for **9** and **10**,** 9** demonstrates both the best enzymatic potency and kinome-wide selectivity.

Given its selectivity and TTBK potency, we comprehensively investigated the potential off-targets of **9** identified in the kinome-wide and 17-membered kinase panel selectivity experiments. We selected 19 total kinases from the S_35_(1 μM) fraction^29^ of the *scan*MAX kinase binding results plus kinases potently inhibited in our 17-membered enzymatic panel (Table S1). We added TAOK2, TTBK1, and TTBK2 for enzymatic follow-up because **9** more potently inhibited TAOK2 than TAOK1 in our enzymatic panel (Table S1), and TTBK1/2 are not in the *scan*MAX panel. All WT human kinases for which there was an available enzymatic assay kinase at Eurofins, RBC, or SignalChem (annotated in Table S2) were evaluated in dose–response format. This only excluded PI5P4Kγ, for which a corresponding enzymatic assay was not available. Evaluation of **9** in the PI5P4Kγ NanoBRET cellular target engagement (CTE) assay revealed it to bind this kinase with an IC_50_ = 580 nM (Table S2)^30^. Examination of this data, captured in Table S2, reveals that, in addition to TTBK1/2, six kinases are potently inhibited by **9** with IC_50_ values < 400 nM, and for four of these kinases (MAP4K5, MYLK4, PIKfyve, and YSK4) IC_50_ values < 100 nM were observed in the respective enzyme assays.

### Structural studies confirm that indolyl pyrimidinamines assume a canonical ATP-competitive binding mode and access the TTBK1 back pocket

Motivated to understand how these potent inhibitors bind to TTBK, we solved TTBK1 co-crystal structures with the four most potent TTBK inhibitors: AMG28, **3**, **9**, and **10**. The aminopyrimidine ring makes two key hydrogen bonds with TTBK1 backbone hinge residues Q108 and Q110. Interestingly, our structures demonstrate that F45 at the tip of the P-loop is highly flexible and can adopt both ‘in’ and ‘out’ conformations. Ring-opened analog **3** induces an ‘in’ conformation of P-loop F45 (Fig. 2B), whereas those that are locked into a ring (AMG28, **9**, and **10**) bind with F45 in an ‘out’ conformation (Fig. 2A). We postulate that the inward movement of F45 stabilizes **3**, which is supported by a thermal shift value that is similar to that of its ring-closed parent, AMG28 (Fig. 2C), and how its TTBK1/2 potency was maintained (Tables 1 and S1).

The solvent-exposed seven-membered ring shared by **9** and **10** makes these analogs very planar, which was similarly observed with **3**, and helps orient the substituent on the alkyne to interact with E77 and F177 in the back pocket. The introduction of an ethyl group in **9** and **10** enabled space filling within the hydrophobic back pocket formed by gatekeeper M107, αC L81, and the DFG ‘in’ F177 that was not achieved by AMG28. This back pocket, which is accessed via displacement of the catalytic lysine to access a narrow channel in TTBK1 formed by M107, K63, L81, and F177 from the DFG motif, is filled by hydrophobic groups on **9** and **10** that enhance their binding versus AMG28 (Fig. 2A). This resulted in an increase in kinase stabilization, which registers as a melting temperature (Tm) shift that is ~ 1.2–1.8 °C higher for **9** and **10** versus AMG28 (Fig. 2C). The trend in thermal shifts was consistent with what we observed in enzymatic assays, with the most potent enzymatic inhibitors resulting in the largest thermal shifts.

### Development of TTBK1 and TTBK2 cellular target engagement assays

We employed the NanoBRET technology to enable CTE assays for TTBK1/2. While cDNA corresponding to the N- and C-terminally NanoLuciferase (NLuc/NL) tagged versions of TTBK1/2 were provided to us by Promega, a screen of their commercially available NanoBRET tracers demonstrated that none provided a suitable assay window. A NanoBRET tracer is an ATP-competitive small molecule ligand validated to bind the kinase target and modified to bear a red-shifted fluorophore. The NLuc-tagged kinase and NanoBRET tracer produce bioluminescence resonance energy transfer (BRET) when the tracer is bound to the kinase active site, and this BRET can be competed away in a dose-dependent manner by ATP-competitive ligands. An IC_50_ value is then calculated and corresponds with engagement of the kinase by a test compound in live cells^31^.

**Figure 3:**
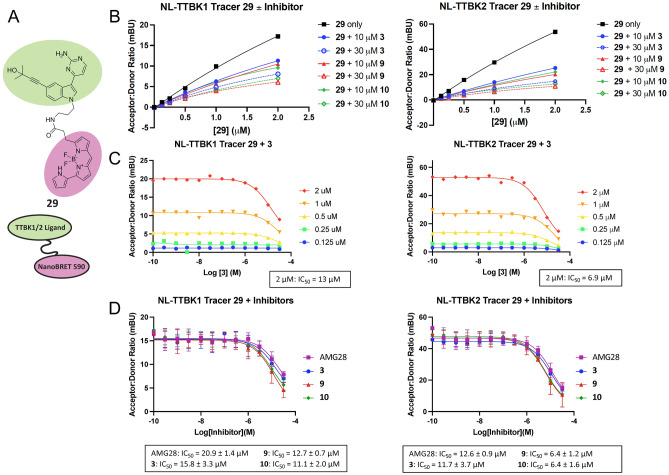
Cell-based NanoBRET assays with TTBK lead inhibitors. (**A**) Structure of tracer **29**. (**B**) Competition experiments between tracer **29** and analog **3**, **9**, or **10** at either 10 or 30 µM for NLuc-TTBK1 and NLuc-TBK2, n = 1. (**C**) Tracer titration results for NLuc-TTBK1 and NLuc-TTBK2 in the presence of analog **3**, n = 1. Calculated IC_50_ values for **3** are shown for each kinase at the recommended working tracer concentration of 2 µM. (**D**) Representative NanoBRET assay curves and corresponding IC_50_ values for active compounds AMG28, **3**, **9**, and **10** in intact HEK293 cells, n = 2. Error is shown and reported as standard error mean (SEM).

To enable a NanoBRET assay, we designed a fit-for-purpose tracer based on analog **3**. This parent was selected based upon its ease of synthesis from a commercially available indole (Fig. S2), its conformational freedom versus ring-constrained analogs, and its broader inhibition profile when screened kinome-wide (S_10_(1 μM) = 0.084), making it a potentially useful NanoBRET tracer for other understudied kinases (Fig. S1). Our co-crystal structure of analog **3** bound to TTBK1 (Fig. 2B) demonstrated that the indole nitrogen is solvent exposed. We opted for N-alkylation at this position to install our linker-appended fluorophore (Tracer **29**, Fig. 3A). A short alkyl linker was chosen based on preliminary experiments with other kinases that supported a propyl chain increases cellular permeability of resultant tracers versus those bearing short and long polyethylene glycol linkers.

Initial experiments aimed to determine which NLuc-tagged variant of TTBK1/2 yielded the best BRET signal and whether the tracer was able to permeate cells. Permeation studies were carried out by treating intact HEK293 cells and digitonin permeabilized HEK293 cells (both transiently transfected with a NLuc-TTBK1/2 construct) in dose–response format with 0.5–2 µM of tracer **29** (Fig. S3). The N-terminally tagged version of both TTBK1/2 yielded an optimal signal in the corresponding assays. Dose-dependent inhibition of the BRET signal was observed in intact and permeabilized cells when treated with tracer **29**.

Before evaluating our TTBK inhibitors, we explored whether tracer **29** could be displaced from TTBK1/2 by its unconjugated parent inhibitor (**3**). This competition experiment was used to confirm that the tracer binds in the same pocket as the parent compound instead of non-specifically or to an allosteric site. As shown in Fig. 3B, for TTBK1/2, the BRET signal was decreased in a dose-dependent manner in the presence of different concentrations of tracer **29** through addition of 10 or 30 µM of parent **3**. Furthermore, a competition experiment using 10 or 30 µM of **9** or **10** also displaced tracer **29** (Fig. 3B).

After completing the competition experiments, we executed tracer titration experiments to determine the optimal working concentration of tracer **29** to use in the assays with NLuc-TTBK1 or NLuc-TTBK2. As shown in Fig. 3C, parent compound **3** was dosed in the presence of increasing concentration of tracer: 0.125–2 µM. For both kinases, we found that 2 µM of tracer **29** afforded the best acceptor:donor ratio and provided the most optimal assay window for the respective NanoBRET assays. The IC_50_ of compound **3** based on these tracer titration experiments is between 6 and 13 µM. Sub-optimal cellular penetrance of the tracer (Fig. S3) may contribute to the observed IC_50_ values.

### Confirmation that indolyl pyrimidinamines engage TTBK1/TTBK2 in live cells

The TTBK inhibitors were profiled in dose–response format using the enabled TTBK1/2 NanoBRET assays in intact and permeabilized HEK293 cells (Table 2). IC_50_ values for the majority of TTBK inhibitors were > 30 µM in the live cell NanoBRET assay, except for AMG28, **3**, **9**, and **10**, which were active in both assays. Two additional analogs (**1** and **5**) demonstrated IC_50_ values < 30 µM in the TTBK2 NanoBRET assay only. AMG28, **3**, **9**, and **10** exhibited IC_50_ values of 11–21 µM in the TTBK1 NanoBRET assay and 6–13 µM in the TTBK2 NanoBRET assay and, consistent with results in Table 1, all analogs demonstrated more efficacious engagement of TTBK2 than TTBK1. TTBK1 enzymatic IC_50_ values of 380–820 nM and TTBK2 enzymatic IC_50_ values of 175–990 nM translated to micromolar values in the respective NanoBRET assays for AMG28, **3**, **9**, and **10**. A potency loss in moving to cells was anticipated and indicates that additional optimization is warranted to improve both enzymatic and cellular potency of the leads.

**Table 2:** NanoBRET and solubility data for Indolyl Pyrimidinamine Library


Compound	Scaffold	n	R	TTBK1 intact cell NB IC_50_ (µM)^[a]^	TTBK1 permeabilized cell NB IC_50_ (µM)^[b]^	TTBK2 intact cell NB IC_50_ (µM)^[a]^	TTBK2 permeabilized cell NB IC_50_ (µM)^[b]^	Kinetic solubility (µM)
**1**	A	1		> 30	2.5	16	3.6	NT^[c]^
**AMG28**	A	2		20.9 ± 1.4^[e]^	2.88 ± 0.26^[e]^	12.6 ± 0.9^[e]^	4.10 ± 0.65^[e]^	NT
**2**	A	3		> 30	19	> 30	17	NT
**3**	B	N/A^[d]^		15.8 ± 3.3^[e]^	2.34 ± 0.31^[e]^	11.7 ± 3.7^[e]^	2.52 ± 0.62^[e]^	133
**4**	A	2		> 30	20	> 30	> 30	NT
**5**	A	2		> 30	7.2	26	9.3	NT
**6**	A	2		> 30	25	> 30	19	NT
**7**	A	2		> 30	> 30	> 30	> 30	NT
**8**	A	2		> 30	> 30	> 30	> 30	NT
**9**	A	2		12.7 ± 0.7^[e]^	2.11 ± 0.43^[e]^	6.40 ± 1.17^[e]^	1.47 ± 0.36^[e]^	50
**10**	A	2		11.1 ± 2.0^[e]^	1.97 ± 0.00^[e]^	6.39 ± 1.59^[e]^	1.39 ± 0.44^[e]^	131
**11**	A	2		> 30	> 30	> 30	> 30	NT

^a^Assay executed in intact HEK293 cells. ^b^Assay executed in permeabilized HEK293 cells. ^c^NT: not tested. ^d^N/A: not applicable. ^e^Data representative of two biological replicates ± SEM.

For both kinases, permeabilizing the cells improved the calculated IC_50_ values compared to those obtained in intact cells. AMG28, **3**, **9**, and **10** exhibited IC_50_ values < 4.1 µM in both NanoBRET assays and IC_50_ values for the weaker TTBK inhibitors (**1**, **2**, **4**, **5**, and **6**) improved as well (Table 2). The TTBK2 bias of the compounds was no longer observed when cells were permeabilized. Curves for the active compounds are included in Fig. 3D (intact cells) and Fig. S3 (permeabilized cells). The left-shifted curves in permeabilized cells suggests an opportunity to improve the cellular penetrance of the analogs in future optimization efforts. To explore whether poor aqueous solubility was contributing to this result, kinetic solubility of **3**, **9**, and **10** was evaluated. Analog **9** was less soluble than **3** and **10**, but all were in an acceptable range (Table 2).

### Analog 10 modulates TTBK downstream signaling

**Figure 4:**
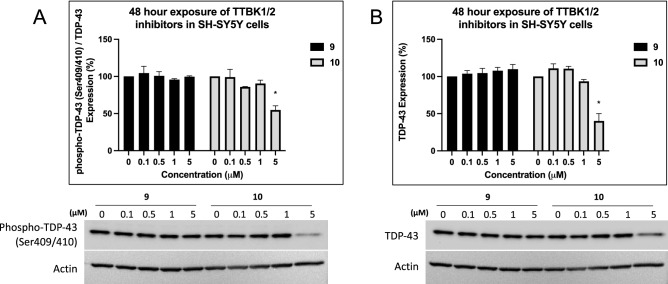
Effects of TTBK lead inhibitors on downstream signaling. (**A**) Western blot analyses of SH-SY5Y cells after 48 h treatment with** 9** or **10**. Quantification of phospho-TDP-43 (Ser409/410, normalized to total TDP-43), n = 3. *control versus 5 µM **10** *p*-value = 0.0017. (**B**) Western blot analyses of SH-SY5Y cells after 48 h treatment with** 9** or **10**. Quantification of total TDP-43, n = 3. *control versus 5 µM **10** *p*-value = 0.0001. Error bars represent SEM. *P*-values were generated using one-way ANOVA with Tukey correction. Unformatted images of blots are included in Fig. S8.

To determine whether our inhibitors impact downstream signaling of TTBK1/2, we analyzed whether **9** and **10** inhibit phosphorylation of TDP-43. TTBK1/2 belong to a group of only a few kinases that have been described to phosphorylate TDP-43^12,23^. We first explored the time-dependent effect of our compounds on TDP-43 phosphorylation in SH-SY5Y neuroblastoma cells. After 24 h treatment, there were no changes in phospho-TDP-43 or (total) TDP-43 protein expression at up to 5 μM of **9**, and only (total) TDP-43 expression decreased (~ 40%) at 5 μM with **10** (Fig. S4). After 48 h treatment (Fig. 4A and B), **9** did not change phospho-TDP-43 or total TDP-43 expression when treated with up to 5 μM. In contrast, 5 μM of **10** decreased phospho-TDP-43 expression by ~ 45% and decreased total TDP-43 expression by ~ 60%. Following treatment for 72 h, 5 μM of **9** slightly decreased (~ 13–16%) phospho- and total TDP-43 expression (data not shown). The concentration at which **10** reduced TDP-43 phosphorylation (5 μM) aligns with its IC_50_ values in the TTBK1/2 NanoBRET assays (6.39–11.1 μM, Table 2).

Exposure of SH-SY5Y cells to ethacrynic acid (EA) is reported to induce TDP-43 phosphorylation by causing cell death via glutathione depletion^23,32^. Following a published protocol, we treated SH-SY5Y cells with 5 μM of either **9** or **10** one hour prior to the addition of 40 μM EA^23^. Cells were harvested after 24 h. We did not observe an enhancement of TDP-43 phosphorylation with EA treatment (Fig. S4). One potential explanation is that our phospho-TDP-43 signal at baseline is robust in control cells. Consistent with the 24 h treatment without EA addition (Fig. S4), total TDP-43 was decreased (~ 44%) by 5 μM of **10**, but phospho-TDP-43 was not affected. Limited toxicity was observed after 24 h treatment with 5 μM of **9** or **10**. Based on the appearance of the cells and experiments carried out in parallel using our PIKfyve probe, we propose that at 5 μM these compounds are potently inhibiting PIKfyve^33^. As shown in Table S2 and Fig. S1, both compounds bind with high affinity to PIKfyve. PIKfyve inhibition leads to disruption of lysosome function that drives cell death^34^. LysoTracker staining overlaps with the enlarged vesicles observed at the 5 μM dose (Fig. S4), which is consistent with PIKfyve inhibitor treatment^33,34^. However, we show that PIKfyve inhibition with our PIKfyve chemical probe and a potent PIKfyve lead inhibitor does not decrease phosphorylated or total TDP-43 (Fig. S5).

### Inhibition of TTBK2 reduces cilia in human stem cells

**Figure 5:**
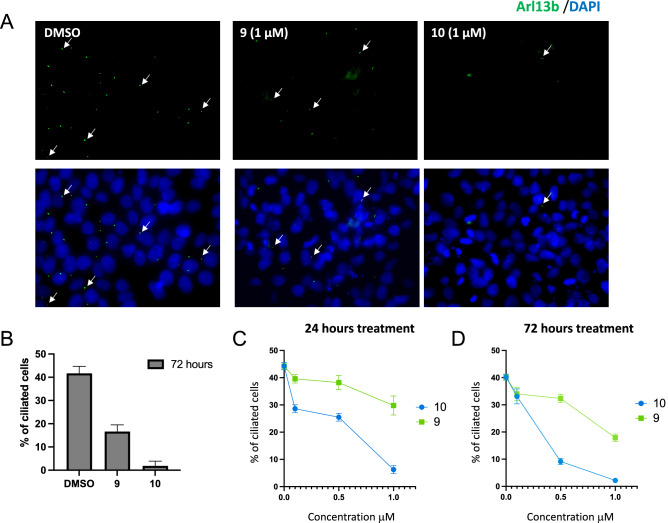
Images and quantification of cilia following treatment of human iPSCs with **9** or **10**. (**A**) Visualization of cilia on human iPSCs following 72 h continuous starvation and treatment with DMSO or 1 μM of either **9** or **10** at 40 × magnification. Arrows are used to highlight the location of some cilia in each panel and a legend of the stains used to visualize cilia and nuclei is placed above the panels. Primary cilia are labeled with the Arl13b antibody (green) and the nuclei with DAPI (blue). (**B**) Quantification of the percentage of ciliated cells per single field in (**A**). Error bars represent the SEM. (**C**, **D**) Quantification of the percentage of ciliated cells averaged from six random visual fields following 24 h (**C**) or 72 h (**D**) of continuous treatment plus starvation and in the presence of different concentrations of **9** or **10**. Error bars represent SEM.

Due to their potency and our interest in studying their impact on cilia, human induced pluripotent stem cells (iPSCs) were treated with **9** or **10** and cilia were visualized. These cells were subjected to two different treatment protocols: 72 h continuous starvation in the presence of inhibitor or 72 h continuous starvation with the inhibitor present for the last 24 h. The influence of concentration and time course of treatment was evaluated. An example of the visual appearance of cells after 72 h continuous starvation in the presence of 1 μM of inhibitor is included in Fig. 5A. Quantification of the percentage of ciliated cells in these single fields is included in Fig. 5B. The percentage of ciliated cells over six visual fields was next calculated for each concentration and timepoint. As shown in Fig. 5C and D, a time- and dose-dependent response of cilia to treatment with both compounds was observed but was more pronounced for **10**. The most robust result was noted following 72 h continuous treatment and at a concentration of 1 μM with either analog.

### TTBK2 genetic knockout in human iPSCs inhibits ciliogenesis

**Figure 6:**
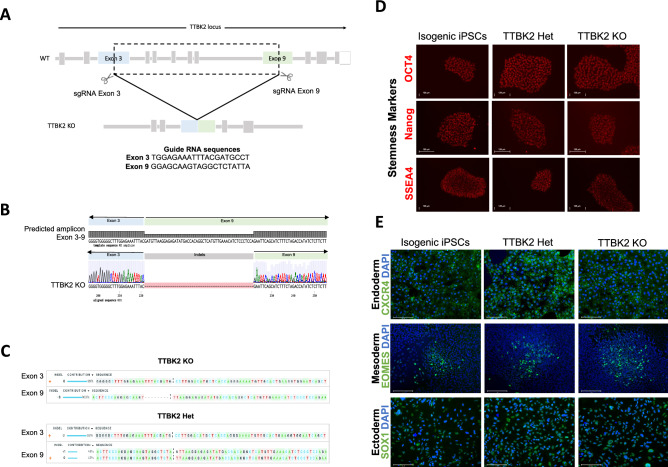
Generation and characterization of TTBK2 knockout iPSCs using CRISPR/Cas9. (**A**) Diagram depicting the TTBK2 locus, the sites on exon 3 and exon 9 targeted by guide RNAs, and the predicted deletion from exon 3–9. Guide RNA sequences for each exon are included below the illustration. (**B**) Graphic showing an alignment of the predicted amplicon resulting from deletion between exon 3 and exon 9 and the Sanger sequence for the TTBK2 KO clone, confirming genomic deletion in one allele. (**C**) Inference of CRISPR Edits (ICE) analysis illustrating the modifications of exon 3 and exon 9 in the homozygous (TTBK2 KO) and heterozygous (TTBK2 Het) edited cells. (**D**) Immunofluorescent staining of stemness markers OCT4 (octamer-binding transcription factor 4), Nanog (Nanog homeobox), and SSEA4 (stage-specific embryonic antigen-4) in isogenic, TTBK2 Het, and TTBK2 KO cells. All markers and their labels are red. Isogenic iPSCs are included for reference. (**E**) Immunofluorescent staining of endoderm (CXCR4, C-X-C motif chemokine receptor 4), mesoderm (EOMES, eomesodermin), and ectoderm (SOX1, SRY-box transcription factor 1) markers illustrating the capability of the homozygous (TTBK2 KO) and heterozygous (TTBK2 Het) edited cells to differentiate into the three germ layers. Isogenic iPSCs are included for reference.

To compare the results observed when treating iPSCs with **9** and **10** with genetically edited cells, we generated a TTBK2 knockout (KO) iPS cell line using the CRISPR/Cas9 system (Figs. 6 and S6). The two-guide RNA system was employed to create a genomic deletion in the TTBK2 gene from exon 3 to exon 9 (Fig. 6A). Two clones were isolated and Sanger sequencing confirmed one homozygous (TTBK2 KO) and one heterozygous (TTBK2 Het) iPSC clone (Fig. 6B,C). No indels were observed on the top four sequence-based predicted potential off-targets of each guide RNA (Fig. S6A–B). Edited iPSCs expressed the stemness markers OCT4, Nanog, and SSEA4, indicating cells remain pluripotent (Fig. 6D). Immunofluorescent staining and the TaqMan hPSC Scorecard assay confirmed the cells capacity to differentiate into endoderm, mesoderm, and ectoderm tissue (Figs. 6E and S6C). These results indicate TTBK2 KO and TTBK2 Het remain pluripotent and retain their ability to differentiate into the three germinal layers.

**Figure 7:**
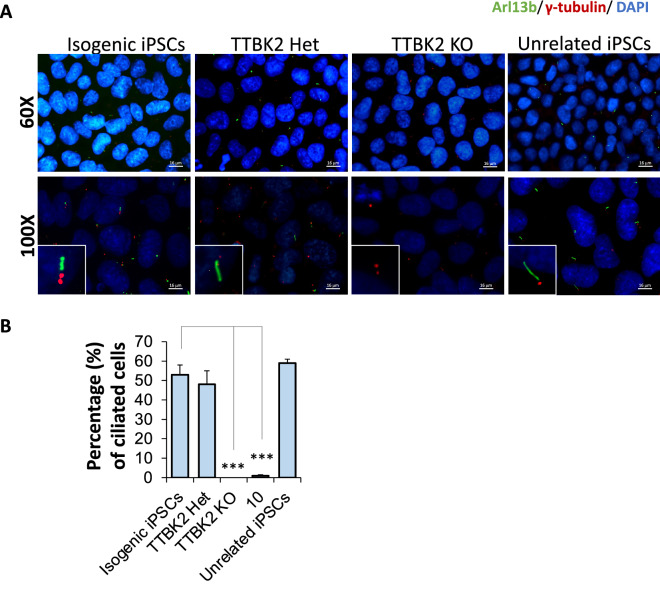
Images and quantification of cilia on human iPSCs compared to TTBK2 KO iPSCs and iPSCs treated with **10**. (**A**) Visualization of cilia on human isogenic iPSCs, TTBK2 Het cells,TTBK2 KO cells, and an unrelated iPSC line (UNCC1001-A) following 72 h starvation. A legend of the stains used to visualize cilia and nuclei is placed above the panels. Primary cilia are labeled with the Arl13b antibody (green), centriole with γ-tubulin (red), and the nuclei with DAPI (blue). Images were taken at 60X (top panels, scale bar 16 µm) or at 100X (bottom panels, scale bar 10 µm). Insets in the bottom panels more clearly show cilia corresponding with each cell line. (**B**) Plot of the percentage of cells expressing cilia averaged from ten random visual fields for human isogenic iPSCs, TTBK2 Het cells, TTBK2 KO cells, and an unrelated iPSC line (UNCC1001-A) versus percentage of cells expressing cilia averaged from six random visual fields following 72 h continuous treatment with 1 µM of **10**. Error bars represent the SEM and *p*-values calculated by comparing the percentage of cells expressing cilia from three different biological experiments using one-way ANOVA. ***p-value < 0.0001.

To compare with inhibitor-treated cells (Fig. 5), cilia were visualized on genetically edited and isogenic control cells. Fig. 7A shows representative images of cells from the isogenic control cells, TTBK2 Het, TTBK2 KO, and an unrelated human iPSC line. These images clearly show that cilia are absent in TTBK2 KO compared to TTBK2 Het and control cells. The lack of cilia expression on TTBK2 KO was observed even in the absence of starvation (Fig. S7). We quantified the percentage of ciliated cells when ten fields were visualized and averaged in these various iPSC lines and plotted those averages versus what was observed after 72 h starvation and continuous treatment with **10**. This graph (Fig. 7B) demonstrates that treatment of iPSCs with **10** phenocopies the TTBK2 KO cells.

## Discussion

The orthogonal data collected for various analogs, together with the co-crystal structures, allows development of structure–activity relationships for the indolyl pyrimidinamine scaffold. A slight bias of our compounds to bind to and inhibit TTBK2 more efficaciously than TTBK1 was observed across the biochemical and cell-based analysis methods. Potency trends for TTBK active compounds was maintained in these orthogonal binding and enzymatic assay formats.

With both a six-membered (**1**) and eight-membered analog (**2**) of AMG28 in which only the size of the ring system was modified, TTBK1/2 potency was significantly altered. The enzymatic data for this small series supports that the seven-membered ring offers enhanced efficacy versus the smaller and larger ring systems. Based on our co-crystal structures (Fig. 2), these rings are solvent-exposed and the analogs are very planar. We propose that the ring system size alters the orientation and trajectory of the alkyne and the ability of the compounds to make key contacts with the back pocket is diminished with analogs **1** and **2** compared to AMG28. When the ring system was removed to yield analog **3**, potency was maintained across enzymatic, binding, and cell-based assays when comparing AMG28 and analog **3** (Tables 1, 2, and S1). Ring-opened analog **3** binds a larger portion of the screenable kinome than AMG28, suggesting that increased conformational flexibility allows binding to a range of kinases.

Subtle structural changes are present when examining analogs **4**–**11**. Comparison of the data in Tables 1, 2, and S1 generated for analogs **9** and **10** versus AMG28 confirms that the portion of the binding pocket that accommodates the alkyne is sensitive to changes in steric bulk. This back pocket is filled by hydrophobic groups on **9** and **10** that enhance their binding versus AMG28 (Fig. 2A). All inhibitors, including AMG28, that protrude into the back pocket produce remarkably higher Tm shift values (Fig. 2C) than TTBK inhibitors that are unable to access the back pocket, including those built upon the AZ2 scaffold (Fig. 1A, ΔTm < 3.5), when tested in parallel^23^. Together the co-crystal structures and potency data suggest that space filling the TTBK1/2 back pocket is a design strategy via which to develop analogs of **9** and **10** with increased kinase stabilization and enhanced inhibitory potency. Examination of other analogs bearing a seven-membered ring (**4**–**8** and **11**) supports that placement of hydrophobic bulk must be very deliberate and branching from the carbon directly attached to the alkyne with two carbon chains is optimal (AMG28, **9**, and **10** versus **4** and **5**). Coupled with this finding is the discovery that the hydroxyl group on the alkyne terminus cannot be moved from this carbon without losses in TTBK potency (AMG28, **9**, and **10** versus **6**–**8**). Finally, analog **11** demonstrates that the hydroxyl group cannot be capped with a methyl group without dramatic losses in activity, indicative of forfeiture of a key interaction and/or interference of the resultant methyl ether with the back pocket.

Analog **9** with two ethyl groups demonstrated the most favorable kinome-wide selectivity, while **10** mimicked the less selective parent compound AMG28, indicating that many kinases do not tolerate this bulkier structural modification. Fifteen fewer kinases are in the S_10_(1 µM) fraction when the methyl (**10**) is swapped for an ethyl (**9**).Our finding that the ethyl substitution on analog **10** resulted in a boost in TTBK potency was echoed by Biogen^24^. Furthermore, BGN18 and BGN31 were scanned in the same DiscoverX *scan*MAX panel at 1 μM and were found to bind with PoC < 10 sixteen and eight kinases, respectively^24^. Many of these off-target kinases are within the S_10_(1 µM) fraction of our TTBK actives (Fig. S1) and were potently inhibited in the MRC panel (Fig. 1B). This side-by-side comparison makes **9**, which binds seven kinases with PoC < 10 at 1 μM (Table 1), the TTBK inhibitor with the lowest kinome-wide selectivity score from these structurally related series.

One additional observation related to analogs **9** and **10** is their differential impacts on reducing TDP-43 phosphorylation after 48 h treatment (Fig. 4). While they showed similar affinity for TTBK1/2 in the respective NanoBRET assays (Table 2) and both impair cilia formation (Fig. 5), only analog **10** significantly inhibited downstream signaling in Fig. 4. Our preliminary result (one replicate) demonstrated that 5 µM of **9** slightly decreased (~ 13–16%) phospho- and total TDP-43 expression compared to control (data not shown). Accordingly, more significant impaired cilia formation was observed following 72 h continuous starvation and treatment with analog **9** versus 72 h continuous starvation and treatment with analog **9** for only 24 h (Fig. 5C). Based on these observations, we propose that analog **9** takes longer to decrease TDP-43 phosphorylation when compared to analog **10**. Additional timepoints would need to be examined to validate this hypothesis via Western blot.

All currently employed cell-based assays used to validate and measure the activity of TTBK inhibitors in cells rely on an indirect measurement, such as tau or TDP-43 phosphorylation^23,24^. There is no commercial or published assay that directly measures engagement of TTBK1/2 by a small molecule in cells. Our TTBK1/2 NanoBRET assays offer direct assessment of binding to TTBK1/2 in live cells. Furthermore, these assays allow evaluation of binding kinetics, compound residence time, and cellular penetrance and can be used to drive iterative medicinal chemistry optimization^35^.

Our results align with literature reports related to the roles of TTBK1/2 in ciliogenesis. Recruited by CEP164, TTBK2 colocalizes with CEP83 at the root of the centriole distal appendage^15,36,37^. Phosphorylation of CEP83 by TTBK2 is essential for the early ciliogenesis steps, including ciliary vesicle docking and CP110 removal from the mother centriole, which initiate assembly of distal appendages to form cilia^36,37^. TTBK2, in concert with other proteins and phosphatidylinositol, engages in remodeling of the distal ends of mother centrioles and basal bodies to allow for activation of microtubule growth and axonemal extension^37^. Other essential ciliogenesis mediators, including CEP164 and CEP97, are characterized TTBK2 substrates as well^15,16,37^. A lack of validated antibodies that bind to centriole proteins has made it difficult to distinguish the precise role of TTBK2 phosphorylation in these ciliogenesis-promoting processes.

Our findings of cilia frequency reduction on TTBK2 KO iPSCs and upon treatment of human iPSCs with our TTBK inhibitors are consistent with reported conditional KO of TTBK2 in adult mice, which results in degenerative cerebellar phenotypes and loss of cilia throughout their brain^38^. In addition, a TTBK2-null mouse mutant lacks primary cilia and exhibits defects in hedgehog signaling. Loss of TTBK2 in this mouse model abolished the recruitment of IFT proteins essential for anterograde and retrograde trafficking as well as axoneme extension and maintenance. These TTBK2-null mice had basal bodies that lacked axonemes^14^. Our next step is to identify an optimized TTBK2 chemical probe to further characterize the role of TTBK2 in mediating ciliogenesis and to explore how TTBK2 loss of function propagates diseases, including SCA11 and others in which cilia abnormalities are pathogenic.

## Conclusions

We have described the design, synthesis, and biological evaluation of a series of indolyl pyrimidinamines as chemical tools to interrogate the functions of TTBK1/2. We have demonstrated several of our analogs are inhibitors of TTBK1/2 and exhibit a slight bias for TTBK2 using three orthogonal assay formats: enzyme, thermal shift, and NanoBRET CTE assays in live cells. The NanoBRET assays described here are the only cellular assay that confirm direct engagement of TTBK1/2 in cells. Solving co-crystal structures of lead analogs with TTBK1 illustrated that the potency of our analogs was derived in part from their ability to access the back pocket of the TTBK1 ATP binding site. Analog **10**, which was confirmed to inhibit downstream TTBK signaling, emerged as a promising tool compound to assess the role of TTBK1/2 in ciliogenesis. This cell-active inhibitor of TTBK2, which binds with high affinity to only a small subset of the screenable human kinome, reduced cilia frequency when dosed in human stem cells. As TTBK2 KO resulted in human iPSCs that completely lacked cilia and analog **10** phenocopied this observation, compound **10** can be used as a tool molecule to characterize TTBK2-mediated pathways in cells. These studies could expand understanding of the roles TTBK2 in ciliogenesis or identify unknown functions of TTBK2. The involvement of cilia in a diverse spectrum of diseases enhances the importance of tools that facilitate the study of cilia biology. Use of analog **10** will help deconvolute the distinct roles of TTBK1/2 and validate additional substrates of these understudied kinases in key disease-propagating pathways.

## Methods

### Cell culture

Human embryonic kidney (HEK293) cells (hypotriploid, female, fetal) were obtained from ATCC and cultured in Dulbecco’s Modified Eagle’s medium (DMEM, Gibco) supplemented with 10% (v/v) fetal bovine serum (FBS, Corning). HEK293 cells were incubated in 5% CO_2_ at 37 °C and passaged every 72 h with trypsin, not allowing them to reach confluency. Human neuroblastoma (SH-SY5Y) cells were obtained from ATCC and cultured in DMEM/F12 medium (Gibco) supplemented with 10% (v/v) fetal bovine serum (FBS, Corning). SH-SY5Y cells were incubated in 5% CO_2_ at 37 °C and passaged every 48 h with trypsin, not allowing them to reach confluency. Human Gibco (TMOi001-A, Thermo Fisher Scientific A18945) and UNCC001-A^39^ iPSCs were cultured on Matrigel-coated dishes in StemFlex medium (Thermo Fisher Scientific A3349401). Cells were passaged twice a week with 0.5 mM EDTA at a 1:10 ratio. Cell media was supplemented with 10 µM Y27632 (Selleckchem S1049) for 24 h after each passage.

### Generation of TTBK2 KO iPSCs

The two-guide RNA (gRNA) target approach was used to create a genomic deletion in the TTBK2 gene. Guide RNAs were designed to target the exon 3 (TGGAGAAATTTACGATGCCT) and exon 9 (GGAGCAAGTAGGCTCTATTA) simultaneously. The ribonucleoprotein (RNP) system was used as described previously^40^. Briefly, 3 × 10^5^ Gibco iPSCs were electroporated with 900 ng of each single gRNA using the Neon Transfection System (Thermo Fisher Scientific). 72 h after electroporation, iPSCs were dissociated into single cells and plated on Matrigel-coated 96-well plates. Single-cell colonies were selected after two weeks, and the genomic DNA was extracted and tested for gene deletion by PCR. Sanger sequencing of exon 3, exon 9, and exon 3–9 of positive clones confirmed cells were successfully edited. Benchling (https://www.benchling.com) was used to predict the potential off-targets of each gRNA based on sequences. The top four potential off-target sites were selected, and Primer-Blast (https://www.ncbi.nlm.nih.gov/tools/primer-blast/) was used to design primers for PCR amplification. Primers were purchased from Eton Biosciences. Genomic DNA was purified from TTBK2 Het and TTBK2 KO cells, amplified by PCR, and Sanger sequencing was performed on the top four potential off-targets of each guide RNA. The TTBK2 KO line was registered in hPSCreg (https://hpscreg.eu) as TMOi001-A-9 and the TTBK2 Het as TMOi001-A-10.

### Characterization of TTBK2 Het and TTBK2 KO clones

Stemness was confirmed by immunofluorescence (IF) staining of pluripotency markers and qPCR with the hPSC Scorecard Panel as previously described^38,40^. Briefly, following KO confirmation iPSCs were plated as colonies, fixed for 15 min with 4% formaldehyde, permeabilized for 15 min with 0.3% Triton X-10, and blocked for 1 h with 5% BSA at room temperature. Cells were incubated with primary antibodies anti- OCT4, Nanog, and SSEA4 at 1:500 dilution overnight. The next day cells were washed with 0.1% Tween, followed by incubation with 1:1000 secondary antibody solution (anti-rabbit AlexaFluor 488 or anti-mouse AlexaFluor 647, both from Life Technologies) in 1% BSA. After three washes, nuclei were counterstained with DAPI (4′,6-diamidino-2-phenylindole, Life Technologies). To confirm trilineage differentiation capabilities, edited iPSCs were differentiated with the STEMdiff Trilineage Differentiation Kit (StemCell Technologies) following the manufacturer’s protocols. Differentiated cells on cover slips where fixed, permeabilized and blocked as described above and incubated with primary antibodies anti-CXCR4 (C-X-C motif chemokine receptor 4, endoderm marker), EOMES (eomesodermin, mesoderm marker) or SOX1 (SRY-box transcription factor 1, ectoderm marker) at 1:500 dilution for two hours. Next, cells were washed with 0.1% Tween followed by incubation with 1:1000 secondary antibody solution (anti-rabbit AlexaFluor 488, Life Technologies) in 1% BSA. After three washes, nuclei were counterstained with DAPI. RNA from non-differentiated and differentiated cells was extracted with RNeasy kit (QIAGEN), reverse transcribed via the high-capacity cDNA reverse transcription kit (Thermo Fisher) and used in the TaqMan hPSC Scorecard Panel (Thermo Fisher) following the manufacturer’s guidelines. The qRT-PCR was carried out using the QuantStudio 7 Flex Real-Time PCR system and analyzed using the hPSC Scorecard Analysis online software (Thermo Fisher)^41^.

### Enzymatic assays

Eurofins kinase enzymatic radiometric assays were executed at the K_m_ value for ATP using a single concentration (1 μM) in duplicate to generate PoC values for TTBK1 and TTBK2 in Table 1 as well for as each kinase listed in Table S1. Eurofins kinase enzymatic radiometric assays were executed at the K_m_ value for ATP in dose–response (9-pt curve) format for TTBK1 and TTBK2, included in Table 1 as enzymatic IC_50_ values, as well as for the majority of kinases in Table S2. Details about the substrate used, protein constructs, controls, and assay protocol for these kinase assays are available at the Eurofins website: https://www.eurofinsdiscoveryservices.com. Reaction Biology Corp. (RBC) radiometric HotSpot kinase assays were carried out at the K_m_ value for ATP in dose–response (10-pt curve) format for MYLK4, RIPK5, and YSK4 in Table S2. Details about the substrate used, protein constructs, controls, and assay protocol for these kinase assays are available at the RBC website: https://www.reactionbiology.com/list-kinase-targets-us-facility. SignalChem developed an ADP-Glo assay to test for enzymatic inhibition of PIKfyve in dose–response (10-pt curve) format in duplicate^33^.

### Kinome-wide analyses

The Eurofins DiscoverX Corporation *scan*MAX assay platform was employed to assess the selectivity of specific analogs at a single concentration (1 µM). As described previously, this assay platform profiles a compound against 403 wild-type human kinases and delivers percent of control (PoC) values^42^. These percent of control values are included in Table 1 and corresponding kinome tree diagrams as well as tables for each analog included in Fig. S1.

### General information for NanoBRET assays

HEK293 cells were transfected with constructs of TTBK1 or TTBK2 tagged with NLuc on the N- or C-terminus as previously described^43^ Constructs for NanoBRET measurements of TTBK1 (NLuc-TTBK1 and TTBK1-NLuc) and TTBK2 (NLuc-TTBK2 and TTBK2-NLuc) were kindly provided by Promega. NLuc orientations used in the respective assays are indicated. Cells were plated in 96-well tissue culture treated plates (Corning 3917) at a cell density of 2 × 10^5^ cells/mL, with a total volume of 100 μL per well in DMEM. After 16 h, the media was aspirated from the plate and replaced with room temperature Opti-MEM without phenol red (Gibco, 100 μL in no tracer wells; 95 μL in tracer only wells; 90 μL in no tracer wells with digitonin; 85 μL in only tracer wells with digitonin). Plates were incubated for 25 min if digitonin was added or for 2 h for the intact cell NanoBRET assay. NanoBRET plates were read after addition of 50 μL of a stock solution (3X) containing NanoBRET NanoGlo substrate (Promega N2161), extracellular NanoLuc inhibitor (Promega N2161) and Opti-MEM. For a 96-well plate the 3X stock solution was prepared with 30 μL of NanoBRET NanoGlo substrate, 10 μL of extracellular NanoLuc inhibitor, and 4960 μL of Opti-MEM without phenol red. Raw milliBRET units (mBU) were read on a GloMax Discover system (Promega) with a donor emission wavelength of 450 nm and an acceptor emission wavelength of 600 nm. mBU were calculated by dividing the acceptor emission values (600 nm) by the donor emission values (450 nm). For NanoBRET studies with TTBK1/2, background corrected mBU were calculated by subtracting the no tracer wells from the tracer containing wells and multiplying by 1000.

### Tracer permeation studies

HEK293 cells were transfected with constructs of TTBK1 or TTBK2 tagged with NLuc on the N- or C-terminus. Tracer **29** (20X) was prepared from a 400 μM stock solution in DMSO with tracer dilution buffer (Promega N291B) and 20% DMSO, with a final assay plate concentration of 1% DMSO. 5 μL of **29** (20X) was added to each well, with exception of the no tracer control wells, with final plate concentrations of 0.5, 1, and 2 μM. 10 μL of digitonin (Fisher, 10X) was added to specific wells to permeabilize the cells. The 10X digitonin stock was prepared by diluting a 40X DMSO solution of digitonin with room temperature Opti-MEM without phenol red, for a final assay plate concentration of 1% DMSO. Plates containing digitonin were incubated for 25 min and non-digitonin containing plates were incubated for 2 h. Two biological replicates each with two technical replicates were plotted in a bar chart with the standard deviation represented as error bars (Fig. S3).

### Tracer competition analyses

HEK293 cells were transfected with NLuc-TTBK1 or NLuc-TTBK2. Compounds **3**, **9**, and **10** were used to compete away the NanoBRET signal produced with tracer **29**. These compounds displace the tracer due to occupancy of the same binding site. Tracer **29** (20X) stock solutions were prepared in tracer dilution buffer (Promega N291B) for final assay plate concentrations of 0.125, 0.25, 0.5, 1, and 2 μM, with a final assay plate concentration of 1% DMSO. 10X stock solutions of compounds **3**, **9**, and **10** were prepared from 10 mM DMSO stock solutions with room temperature Opti-MEM without phenol red, for final assay plate concentrations of 10 μM and 30 μM. 5 μL of **29** (20X) was added to each well, with exception of the no tracer control wells, and 10 μL of 10X solutions of compounds **3**, **9**, and **10** were added to wells for tracer competition. One biological replicate was plotted in GraphPad Prism with [inhibitor] vs. response (three parameters) and included in Fig. 3B.

### Tracer titration experiments

HEK293 cells were transfected with NLuc-TTBK1 or NLuc-TTBK2. Compound **3** was tested in 12-point dose–response format with a top concentration of 30 μM. Tracer **29** (20X) stock solutions were prepared in tracer dilution buffer (Promega N291B) and 20% DMSO, for final assay plate concentrations of 0.125, 0.25, 0.5, 1, and 2 μM. The final assay plate concentrations included 1% DMSO. 5 μL of **29** (20X) was added to each well, with exception of the no tracer control wells, and 10 μL of 10X threefold diluted solutions of compound **3** was added to wells. One biological replicate was plotted in GraphPad Prism with log[inhibitor] vs. response (three parameters) and included in Fig. 3C.

### NanoBRET assays

NanoBRET assays were carried out in dose–response format as described previously^43^. NLuc orientations used in the respective assays are as follows: NLuc-TTBK1, NLuc-TTBK2, and PIP4K2C-NLuc (for PI5P4Kγ). Based on the tracer titration results, assays were carried as described by the manufacturer using 2.0 μM of tracer **29** for TTBK1, 2.0 μM of tracer **29** for TTBK2, and 0.063 μM of tracer K8 for PI5P4Kγ. 20X stock solutions of the respective tracers were prepared in tracer dilution buffer (Promega N291B) and 20% DMSO for final assay plate concentration of 2 μM (TTBK1/2) or 0.063 μM (PI5P4Kγ), with a final assay plate concentration of 1% DMSO. For TTBK1 and TTBK2, compounds were tested in 12-point dose–response format with a top concentration of 30 μM (data in Table 2). Data are reported as IC50 ± standard error mean (SEM). For PI5P4Kγ, **9** was tested in 12-point dose–response format with a top concentration of 10 μM (data in Table S2). One biological replicate was plotted in GraphPad Prism with log[inhibitor] vs. response (three parameters). Plots of TTBK1/2 data are included in Figs. 3D and S3.

### SH-SY5Y western blot analyses and lysosomal imaging

SH-SY5Y cells were exposed to compound for the time period indicated, with or without co-treatment with ethacrynic acid (EA), in Figs. 3/S4/S5 before harvesting lysates. 0.05% DMSO was used as a control. Lysates were collected in RIPA, sonicated, and quantified using BCA assay. 20 μg of protein was run on each lane. Blots were probed with 1:5000 phospho-TDP-43 (Proteintech #22309-1-AP) or 1:10,000 TDP-43 (Proteintech #10782-2-AP). 1:5000 Actin (Sigma #A2228) was used as a loading control on each blot. TDP-43 protein was normalized to actin loading control only. Phospho-TDP-43 was normalized to actin, and then normalized to TDP-43 to account for differences in (total) TDP-43 expression between doses. For imaging of lysosomes (Fig. S4), SH-SY5Y cells were plated in 35 mm glass bottom dishes and treated for 24 h with either 0.05% DMSO (control) or 5 µM of **9** or **10**. After 24 h, 50 nM LysoTracker Red DND-99 (ThermoFisher) was added to each dish, and they were incubated for 30 min at 37 °C. Live cells were imaged at 40X magnification using a microscope equipped with a camera. ImageJ software was utilized for image generation.

### Kinetic solubility

Analysis of kinetic solubility was carried out by Analiza, Inc using 10 mM DMSO stocks of compounds in phosphate buffered saline solution (PBS) at pH 7.4 as described previously^28^. Calculated solubility values in Table 2 have been corrected for background nitrogen present in the DMSO and media.

### Crystallization and structure determination

Recombinant TTBK1 (aa 13–320) co-expressed with lambda phosphatase in *E. coli* Rosetta as a His-Sumo-tagged protein was initially purified by Ni^2+^-affinity chromatography, and the tag was cleaved by SENP1 protease treatment. The cleaved proteins were further purified by size exclusion chromatography and stored in buffer composed of 25 mM HEPES pH 7.5, 250 mM NaCl, 0.5 mM TCEP, and 10% glycerol. The kinase at 10 mg/mL was mixed with inhibitors at 1 mM, and the complexes were crystallized using sitting drop vapor diffusion method at 20 °C and the condition containing 14–23% PEG 3350, 0.2 M sodium acetate pH 7.0, and 0.1 M tris pH 7.5–9.0. Crystals were cryo-protected with mother liquor supplemented with 22% ethylene glycol. Diffraction data collected at Swiss Light Source were processed and scaled using XDS^44^ and Aimless^45^, respectively. The structures were solved by molecular replacement using Phaser^46^ and the coordinate of TTBK1 (PDB code: 7Q8W^23^). Model rebuilding was performed using COOT^47^ and refinement via Refmac5^48^. Solved co-crystal structures are included in Fig. 2 and the data collection and refinement statistics are summarized in Table S3.

### Thermal shift assays

Either the TTBK1 or TTBK2 kinase domain at 2 µM in 10 mM HEPES pH 7.5 and 500 mM NaCl was mixed with the inhibitors at 10 µM in the presence of SyPRO orange dye (Invitrogen). A Real-Time PCR Mx3005p machine (Stratagene) was used to measure the fluorescence spectrum. The Tm shift assays and data evaluation for melting temperatures were performed according to the previously described protocol^49^.

### Cilia imaging on iPSCs

Human iPSCs (1 × 10^4^ cells/well) were plated in Matrigel-coated black 96 well plates in culture medium supplemented with 10 µM Y27632 and incubated for two hours at 37 °C and 5% CO_2_. Then, Y27632 was removed, and starvation was induced by not changing the culture medium for the next 72 h. For TTBK inhibitor treatments of human iPSCs, **9** or **10** was introduced at concentrations of 0.1 µM, 0.5 µM or 1 µM when the medium change was performed to remove Y27632 (for 72 h continuous treatment) or after 48 h of starvation (for 24 h continuous treatment). After the starvation and co-treatment period, cells were fixed for 5 min with 4% formaldehyde at room temperature, followed by 5 min with 100% methanol at − 20 °C, and then permeabilized for 15 min with 0.3% Triton X and blocked with 5% BSA at room temperature. Primary antibodies, anti-Arl13b (Abcam ab153725) and anti-γ-tubulin (Sigma T6557), were used at 1:500 dilution for 2 h at room temperature. Secondary antibodies (anti-rabbit AlexaFluor 488 and anti-mouse AlexaFluor 647, both from Life Technologies) were used at 1:1000 dilution for 30 min at room temperature. Nuclei were counterstained with DAPI (Sigma) for 5 min. Images were taken using the built-in camera of the Life Technologies EVOS FL microscope. Both GFP and DAPI EVOS Light Cubes were used for cilia and nuclei detection. Image quantification was done using ImageJ Fiji by modifying the threshold to select the desired stained phenotype and then minimizing noise and analyzing particles. Six to ten images of random fields were quantified for each sample and results were expressed as the percentage of cells expressing primary cilia.

### Statistical analyses

The statistical methods used have been indicated under the figures in which error bars or statistics are included. Replicate numbers indicate the number of biological replicates analyzed to generate the summary figures. GraphPad Prism 8.2.0 software was used for analyses unless otherwise indicated in figure legends or experimental protocols.

## Supplementary Information

Supplementary Information.

## Data Availability

The data generated or analyzed during this study are included in this published article (and its Supplementary Information file). All compounds and reagents generated are available from the corresponding author. The authors confirm that the manuscript complies with the relevant digital image and integrity policies.
