# Supplementary material for: Modulation of tau tubulin kinases (TTBK1 and TTBK2) impacts ciliogenesis
Source: Sci Rep. 2023 Apr 14;13:6118. doi: 10.1038/s41598-023-32854-4 (PMC10104807; doi:10.1038/s41598-023-32854-4)
Supplement: Supplementary file 1 — Supplementary Information. [file 41598_2023_32854_MOESM1_ESM.pdf]

Supporting Information

Modulation of Tau Tubulin Kinases (TTBK1 and TTBK2) Impacts Ciliogenesis

Frances M. Potjewyd[1], Ariana B. Marquez[2], Apirat Chaikuad[3,4], Stefanie Howell[1], Andrea S. Dunn[5], Alvaro A. Beltran[2,6], Jeffery L. Smith[1], David H. Drewry[1,7], Adriana S. Beltran[2,8], Alison D. Axtman[*,1]

[1] Structural Genomics Consortium, UNC Eshelman School of Pharmacy, University of North Carolina at Chapel Hill, Chapel Hill, NC, 27599, USA

[2] Human Pluripotent Cell Core, University of North Carolina at Chapel Hill, Chapel Hill, NC 27599, USA

[3] Institute of Pharmaceutical Chemistry, Goethe University Frankfurt, Max-von-Laue-Str. 9, D-60438 Frankfurt, Germany

[4] Structural Genomics Consortium, Buchmann Institute for Life Sciences, Goethe University Frankfurt, Max-von-Laue-Strabe 15, D-60438 Frankfurt, Germany

[5] Department of Computer Science, University of North Carolina at Chapel Hill, Chapel Hill, NC 27599, USA

[6] Neuroscience Center, University of North Carolina at Chapel Hill, Chapel Hill, NC 27599, USA

[7] UNC Lineberger Comprehensive Cancer Center, School of Medicine, University of North Carolina at Chapel Hill, Chapel Hill, NC, 27599, USA

[8] Department of Genetics, University of North Carolina at Chapel Hill, Chapel Hill, NC 27599, USA

* Corresponding author e-mail: alison.axtman@unc.edu

Table of Contents

Table S1	S2
Table S2	S2
Table S3	S3
Figure S1	S4
Figure S2	S5
Figure S3	S6
Figure S4	S7
Figure S5	S8
Figure S6	S9
Figure S7	S10
Figure S8	S11
Figure S9	S12
Figure S10	S13
Chemistry experimental details	S14–S15
Compound purity traces and spectra	S16–S20
References	S21

Table S1. Enzymatic Profiling of Indolyl Pyrimidinamine Library in Targeted Kinase Panel.

Compound	PoC at 1 μM[a]																
	HIPK1[b]	MAP4K5	MARK3[b]	MARK4[b]	PAK3[b]	PAK4	PAK5[b]	PAK6[b]	TAOK1[b]	TAOK2[b]	TAOK3	TSSK1[b]	TSSK2[b]	TSSK3[b]	TSSK4[b]	TTBK1[b]	TTBK2[b]
**1**	76	3	56	53	86	58	48	51	5	28	11	48	88	11	55	78	43
**AMG28**	67	1	12	18	86	8	3	6	2	26	9	10	57	1	70	50	20
**2**	96	9	76	86	100	67	87	77	65	83	78	82	76	120	77	92	69
**3**	87	1	46	43	104	30	31	40	7	12	20	30	78	5	78	43	24
**4**	77	2	52	48	81	21	27	18	11	33	20	46	82	24	75	78	64
**5**	69	-1	27	37	89	10	8	13	2	8	9	8	55	13	86	68	72
**6**	30	1	31	37	89	22	26	36	1	9	11	57	91	36	103	91	76
**7**	56	3	84	84	110	31	39	40	24	21	28	98	93	34	103	86	104
**8**	84	6	83	73	117	66	77	82	22	55	44	73	77	59	94	85	103
**9**	124	2	77	60	103	36	60	40	36	31	43	38	76	5	102	41	17
**10**	89	1	48	39	117	12	17	14	9	14	18	16	58	-3	88	37	18
**11**	96	9	104	103	100	91	88	92	73	44	89	89	86	75	101	117	110

[a] Compounds tested at a single concentration (1 μM) in duplicate. [b] IDG kinase

Table S2. Enzymatic and NanoBRET Follow-up of Kinases Potently Inhibited by 9.

Kinase[a]	DiscoverX PoC value	Assay format	IC_{50} (nM)
CHK2	22	Enzymatic	4100
CLK2	27	Enzymatic	2800
CRIK	30	Enzymatic	>10000
CSNK1D	21	Enzymatic	420
CSNK1E	13	Enzymatic	820
MAP4K5	21	Enzymatic	6.0
MKK4	3.5	Enzymatic	>10000
MYLK4[b]	2.4	Enzymatic	90
PIKfyve[c]	0	Enzymatic	1.8
PI5P4Kγ[d]	15	NanoBRET	580
PIP5K1C	0	Enzymatic	2100
PKD3	0	Enzymatic	1100
RIPK5[b]	4.1	Enzymatic	1300
STK16	28	Enzymatic	300
TAOK1	24	Enzymatic	780
TAOK2	66	Enzymatic	860
TSSK3	24	Enzymatic	240
TTBK1	ND[e]	Enzymatic	380
TTBK2	ND	Enzymatic	180
YSK4[b]	1.1	Enzymatic	51

[a] All kinase assays executed at Eurofins unless noted. [b] Evaluated by RBC. [c] Evaluated by SignalChem. [d] Evaluated at SGC-UNC. [e] Not determined.

Table S3. Crystallographic Data Collection and Refinement Statistics.

Complex	TTBK1-AMG28	TTBK1-3	TTBK1-9	TTBK1-10
**PDB accession code**	7ZHN	7ZHO	7ZHP	7ZHQ
Data Collection				
Resolution[a] (Å)	48.40-1.85 (1.91-1.85)	47.48-2.08 (2.15-2.08)	48.47-1.80 (1.86-1.80)	48.33-1.80 (1.86-1.80)
Spacegroup	*C* 2	*P* $2_1 2_1 2_1$	*C* 2	*C* 2
Cell dimensions	*a* = 171.8, *b* = 39.6, *c* = 49.7 Å<br>*α, γ* =90.0°, *β* = 103.2°	*a* = 39.4, *b* = 49.4, *c* = 171.1 Å<br>*α, β, γ* =90.0°	*a* = 172.3, *b* = 39.8, *c* = 49.8 Å<br>*α, γ* =90.0°, *β* = 103.2°	*a* = 171.3, *b* = 39.5, *c* = 49.7 Å<br>*α, γ* =90.0°, *β* = 103.2°
No. unique reflections[a]	28,144 (2,758)	20,721 (1,914)	30,838 (2,992)	30,388 (2,961)
Completeness[a] (%)	100.0 (99.9)	99.0 (96.9)	100.0 (100.0)	100.0 (100.0)
I/σI[a]	13.4 (1.9)	12.5 (2.1)	15.2 (2.0)	13.4 (2.0)
R_{merge}[a]	0.086 (0.884)	0.145 (0.998)	0.071 (0.852)	0.085 (0.780)
CC (1/2)	0.999 (0.773)	0.998 (0.740)	0.999 (0.817)	0.999 (0.829)
Redundancy[a]	6.7 (6.4)	11.7 (9.5)	6.7 (6.8)	6.7 (6.4)
Refinement				
No. atoms in refinement (P/L/O)[b]	2,418/ 25/ 225	2,419/ 22/ 172	2,399/ 27/ 238	2,415/ 26/ 224
B factor (P/L/O)[b] ($Å^2$)	32/ 21/ 38	37/ 30/ 39	33/ 23/ 39	27/ 19/ 33
R_{fact} (%)	19.0	19.6	17.7	17.4
R_{free} (%)	21.5	25.8	21.8	21.2
rms deviation bond[c] (Å)	0.014	0.012	0.013	0.014
rms deviation angle[c] (°)	1.5	1.3	1.4	1.5

[a] Values in brackets show the statistics for the highest resolution shells. [b] P/O indicate protein, ligand and others (water and solvent molecules), respectively. [c] rms indicates root-mean-square.

A

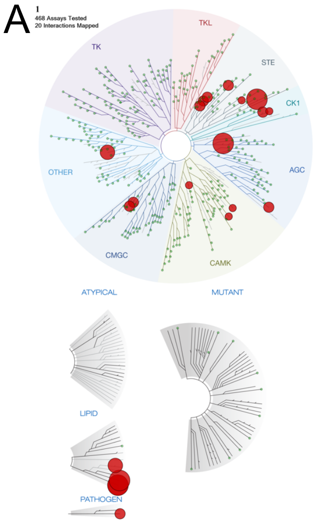

Kinase	% control at 1000nM
YSK4	0
PIKFYVE	0
CIT	0
PIP5K2C	0
HASPIN	0.3
PIP5K1C	0.5
TAOK1	1.2
TAOK3	1.5
TAOK2	1.5
PKNB(M.tuberculosis)	1.7
MAP4K2	2
CSNK1D	3
PRKCE	3.1
CDK11	4.7
CDK8	4.8
CSNK1E	6.2
MEK4	7.3
RPS6KA4(Kin.Dom.2-C-terminal)	7.3
CHEK2	7.9
MYLK4	9.4

B

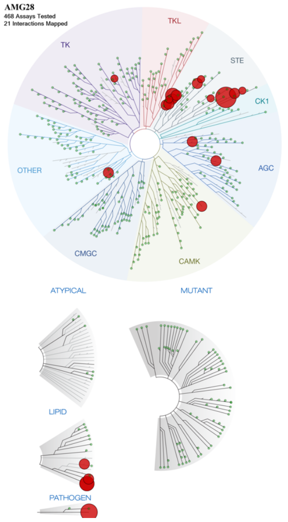

Kinase	% control at 1000nM
YSK4	0
PKNB(M.tuberculosis)	0.1
PAK7	0.3
PIKFYVE	0.4
MAP4K2	1.3
CIT	1.3
TAOK3	1.8
RIPK5	2.2
MYLK4	2.8
PAK6	2.9
PIP5K2C	3.1
PIP5K1C	3.1
TAOK1	3.2
TAOK2	3.5
MAP3K2	3.7
PRKX	4.3
PAK4	5.7
MAP4K5	6.6
JAK2(JH1domain-catalytic)	6.9
MAP3K3	7.2
MEK4	8.6

C

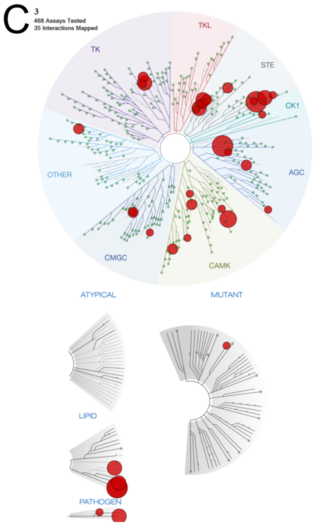

Kinase	% control at 1000nM
YSK4	0
PIKFYVE	0
CIT	0
RPS6KA4(Kin.Dom.2-C-terminal)	0.1
PIP5K2C	0.2
PAK7	0.4
TAOK3	0.4
PIP5K1C	0.4
JAK2(JH1domain-catalytic)	0.5
MAP3K2	0.8
PKNB(M.tuberculosis)	0.9
TAOK2	1.3
PRKD3	1.3
MAP4K2	1.6
SNARK	1.7
JAK3(JH1domain-catalytic)	1.8
TAOK1	2.1
PRKX	3.5
AURKB	3.7
CDC2L1	4.3
TYK2(JH1domain-catalytic)	6.3
BRAF(V600E)	6.5
CDC2L2	6.6
PAK6	6.7
PRKCE	6.8
MAP3K3	7.6
PFCDPK1(P.falciparum)	7.8
TSSK3	8.1
PAK4	8.3
PRKD2	8.3
CSNK1D	8.4
RPS6KA5(Kin.Dom.2-C-terminal)	8.9
GRK2	9.1
NIK	9.4
HIPK1	9.4

D

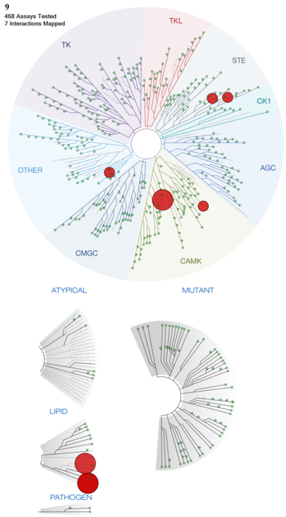

Kinase	% control at 1000nM
PIKFYVE	0
PIP5K1C	0
PRKD3	0
YSK4	1.1
MYLK4	2.4
MEK4	3.5
RIPK5	4.1

E

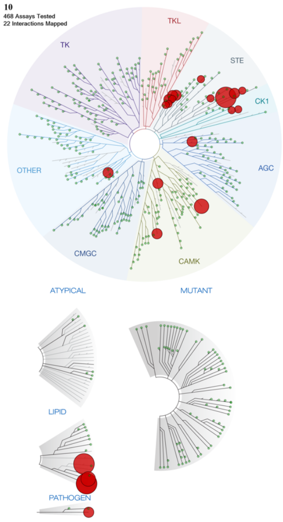

Kinase	% control at 1000nM
PIKFYVE	0
PIP5K1C	0
YSK4	0
MYLK4	0.6
PIP5K2C	0.6
PAK7	1.8
MAP3K2	2.2
CIT	2.4
RIPK5	2.4
TSSK3	2.4
TAOK1	3.1
PAK4	4.3
CHEK2	4.4
PKNB(M.tuberculosis)	4.4
MEK4	5.5
MAP3K3	6.1
PAK6	6.7
TAOK3	7.8
TAOK2	7.9
CSNK1D	8.2
CSNK1E	8.9
MAP4K5	9.6

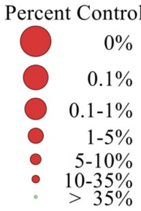

Figure S1. Kinases that Bind with PoC <10 at 1 μM in *scan*MAX Assay Panel at DiscoverX, Related to Selectivity Data in Table 1

(A) **1**, (B) AMG28, (C) **3**, (D) **9**, and (E) **10**. The TREE*spot* kinase interaction mapping software from DiscoverX to prepare the kinome trees (http://treespot.discoverx.com).

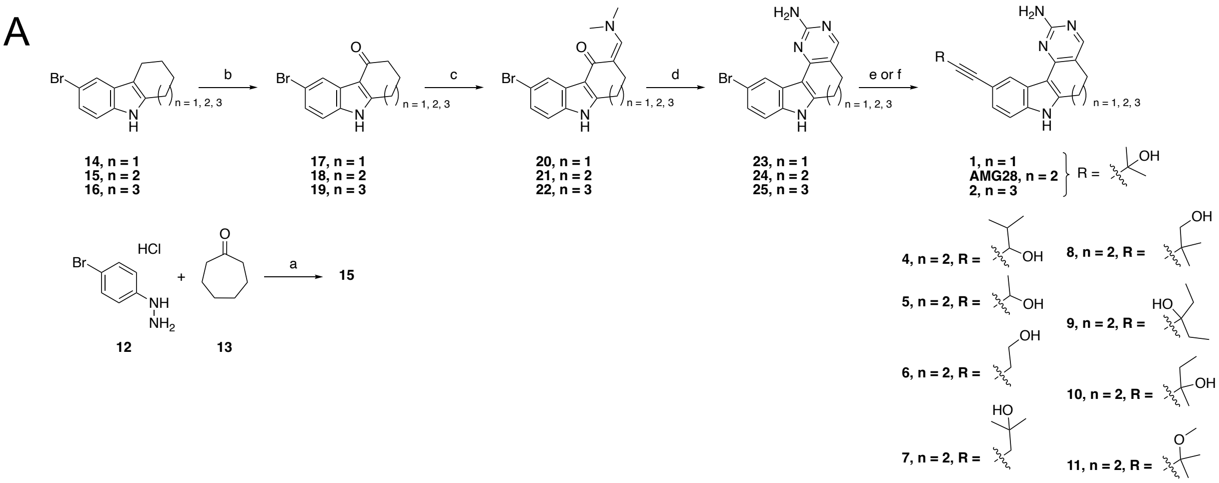

Reagents and conditions: (a) AcOH, 50 °C to 120 °C, 3 h, 70%; (b) DDQ, THF/water, 0 °C, 45 min, 53–87%; (c) Bredereck's reagent, toluene or neat, 110 °C, 3–12 h; (d) NaOMe, guanidine hydrochloride, iPrOH, 100 °C, 12 h, 12–38% over 2 steps; (e) For **1**, **2**, **4**, and AMG28: Alkyne R, TEA, $Pd(PPh_3)_4$, CuI, DMF or DMA, 100 °C, 12 h, 1.6–19%; (f) For **5**–**11**: Alkyne R, DIPA, $PdCl_2(PPh_3)_2$, CuI, propanol, 100 °C, 12 h, 2.4–15%.

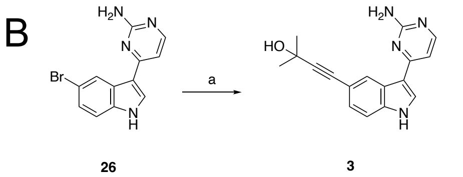

Reagents and conditions: (a) 2-methylbut-3-yn-2-ol, TEA, $Pd(PPh_3)_4$, CuI, DMA, 100 °C, 12 h, 4.4%.

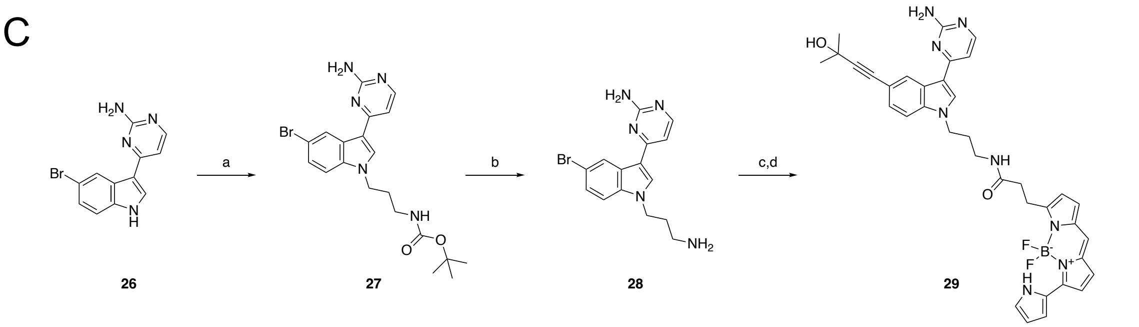

Reagents and conditions: (a) $Tos(CH2)_3NHBoc$, Cs_2CO_3, DMF, 50 °C, 16 h, 35%; (b) TFA, DCM, 2 h, 95%; (c) 2-methylbut-3-yn-2-ol, $Pd(PPh_3)_4$, diisopropylamine, CuI, 80 °C, 16 h; (d) NanoBRET590 SE, DIPEA, DMF, 30 min, 0.9% over two steps.

Figure S2. Schemes Corresponding to Synthesis of all Analogs and Tracer 29.
(A) Route and reagents used to prepare analogs **1**, **2**, **4**–**11**, and AMG28. (B) Route and reagents used to prepare analog **3**. (C) Route and reagents used to prepare tracer **29**.

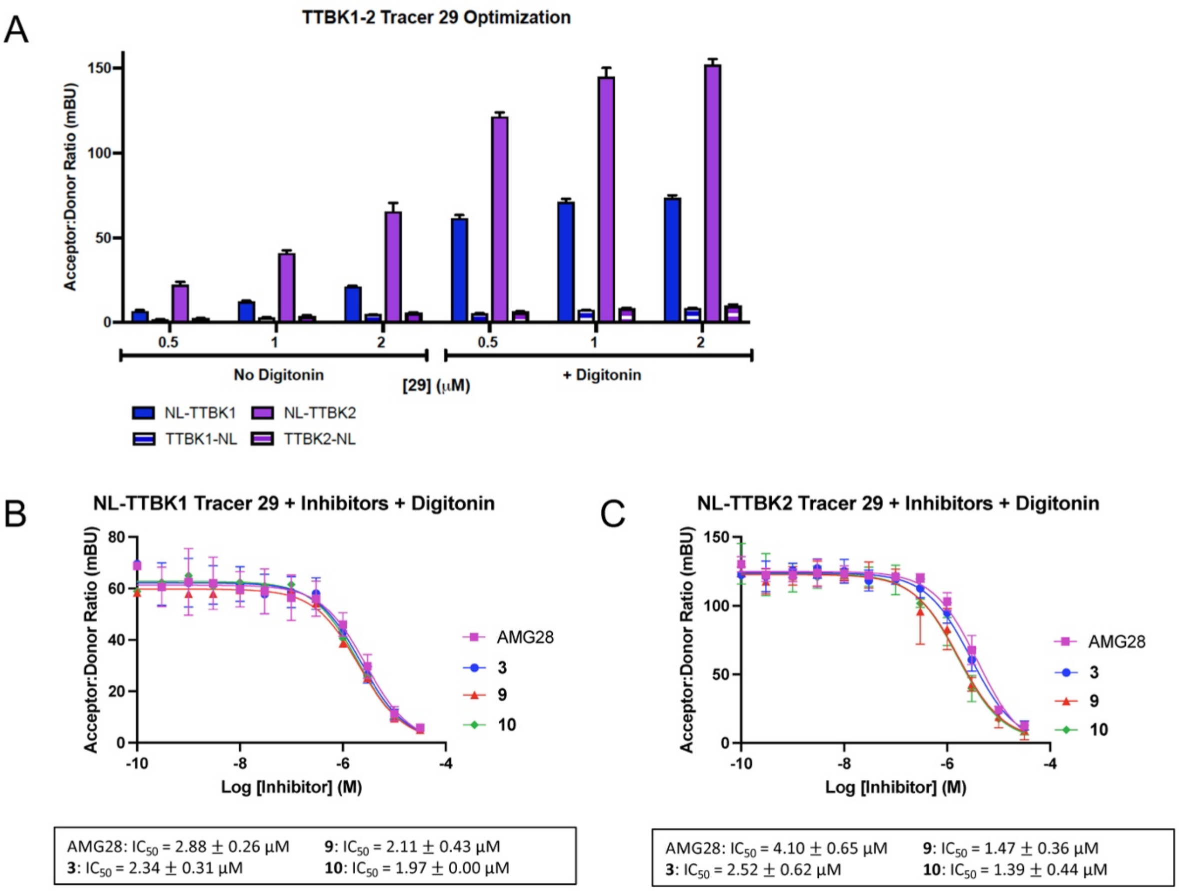

Figure S3. NanoBRET Data Exploring Cell Permeability of Tracer 29.
(A) Permeation studies for tracer **29** in the presence and absence of digitonin and identification of the optimal NLuc-tagged construct for TTBK1 and TTBK2, n = 2. Error bars represent standard deviation.
(B) Representative NLuc-TTBK1 NanoBRET assay curves and corresponding IC_{50} values for active compounds AMG28, **3**, **9**, and **10** in permeabilized HEK293 cells, n = 2. Error is reported as standard error mean (SEM) (C) Representative NLuc-TTBK2 NanoBRET assay curves and corresponding IC_{50} values for active compounds AMG28, **3**, **9**, and **10** in permeabilized HEK293 cells, n = 3. Error is reported as SEM.

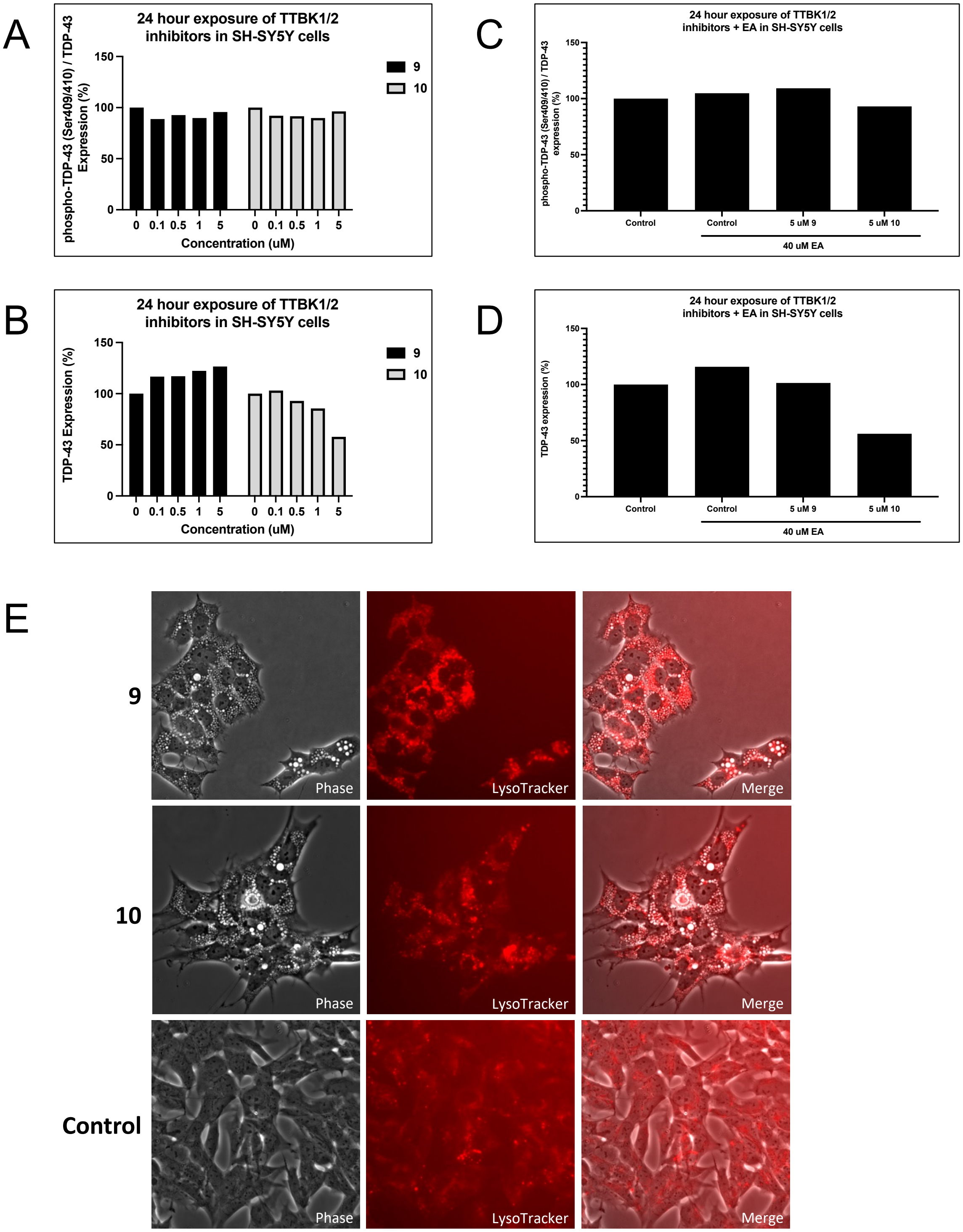

Figure S4. Western blot and Imaging Analyses of SH-SY5Y cells after 24 h Treatment with 9 or 10 with and without the Addition of EA.
(A) Quantification of phospho-TDP-43 (Ser409/410, normalized to total TDP-43) after treatment in dose-response, n = 1. (B) Quantification of total TDP-43 after treatment in dose-response, n = 1. (C) Quantification of phospho-TDP-43 (Ser409/410, normalized to total TDP-43) after treatment with 5 μM compound and 40 μM EA, n = 1. (D) Quantification of total TDP-43 after treatment with 5 μM compound and 40 μM EA, n = 1. (E) 50 nM LysoTracker was added after 24 h treatment with 5 μM **9** or **10** and imaged at 40X. Unformatted images of blots are included in Figure S9.

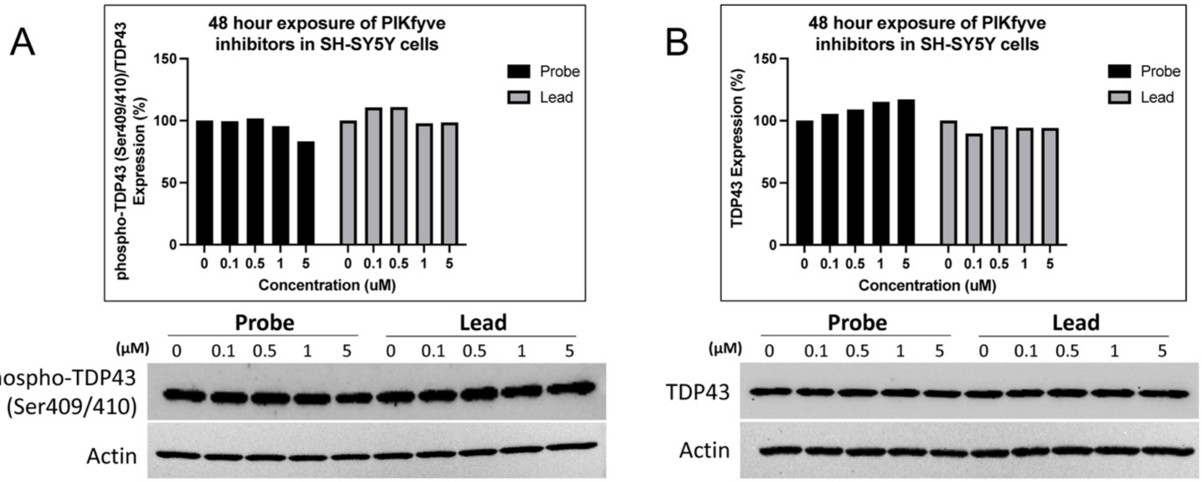

Figure S5. Western blot Analyses of SH-SY5Y cells after 48 h Treatment with PIKfyve chemical probe and PIKfyve lead compound.

(A) Western blots and quantification of phospho-TDP-43 (Ser409/410) expression, normalized to total TDP-43) after treatment in dose response, n = 1. (B) Western blots and quantification of total TDP-43 after treatment in dose response, n = 1. Corresponding compound numbers reported in Drewry et al are PIKyve lead compound = **8** and PIKfyve chemical probe = **17**.[1] Unformatted images of blots are included in Figure S10.

A

ID	Sequence	PAM	Score %	Gene	Chromosome	Strand	Position	Mismatches	On-target	PCR primer FW	PCR Primer RV	Indels
Exon 3												
**ON**	**TGGAGAAATTTACGATGCCT**	**TGG**	**100**	**ENSG00000128881**	**chr15**	**-1**	**42872716**	**0**	**TRUE**	**TGTGGATAAACCTGTGGGGAA**	**GCGTATTTATACCCGGCTGA**	**YES**
OT1	TTGGGACCTTTACGATGCCT	TGG	1		chr6	1	166141774	4	FALSE	TCCCACCTGGCATCCAGTCCTG	GACTTGCATTGGCCCAGGTGCT	NO
OT2	TGTAAAATCTACGATCCCT	CAG	0.7	ENSG00000125740	chr19	-1	45474938	4	FALSE	GCGAGCCGTTGAATTGGAAA	GGCAACAGTGCAGAACCAAG	NO
OT3	TGGCATAATTTACGATTCCT	TGG	0.4	ENSG00000242634	chr15	1	67232672	4	FALSE	CGGCTGTCTGAGGATTGACC	TCCTTTGGCTTTTTGCCAGC	NO
OT4	GGGAGAAGTTTGAGATGCCT	TGG	0.3	ENSG00000162747	chr1	-1	161631014	4	FALSE	GCGCCACTGTCAACACAAATC	TTTCAAGAAAAGGAAACTGGCAGG	NO
Exon 9												
**ON**	**GGAGCAAGTAGGCTCTATTA**	**AGG**	**100**	**ENSG00000128881**	**chr15**	**-1**	**42810724**	**0**	**TRUE**	**CTGATGCAGAACTCTGTGCTAC**	**TGTCAGCAGTTCTATACGCCG**	**YES**
OT5	GAACAATGTAGGCTCTATTA	AGG	1		chr3	-1	104426814	4	FALSE	CTTACACAAGCACAAAAGGCCC	GTGCTTTACAGTTCATGCTTCCA	NO
OT6	GCAGAAACTAGGCTCTGTTA	TGG	0.7		chr3	1	28426372	4	FALSE	ACCAGAAAGATAAAACTCTCCACAA	ACTTTACTTGGGGCTTTTCCCT	NO
OT7	GAAGCATTTAGGCTCTATCA	AGG	0.4	ENSG00000171862	chr10	1	87865957	4	FALSE	AAATGCTGGCAAGAGGGGTT	GCGGGTCCAGTTTAGCCTT	NO
OT8	GGATCAAGAAGATTCTATTA	AAG	0.2	ENSG00000271389	chr3	-1	80163533	4	FALSE	GGGGTCAAGCTGTGCTCTAT	GCCTCTGCAACATCCTCTCT	NO

B

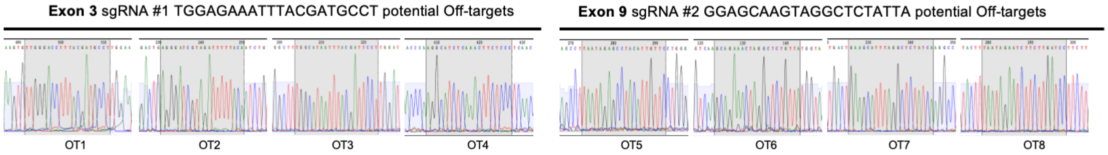

C

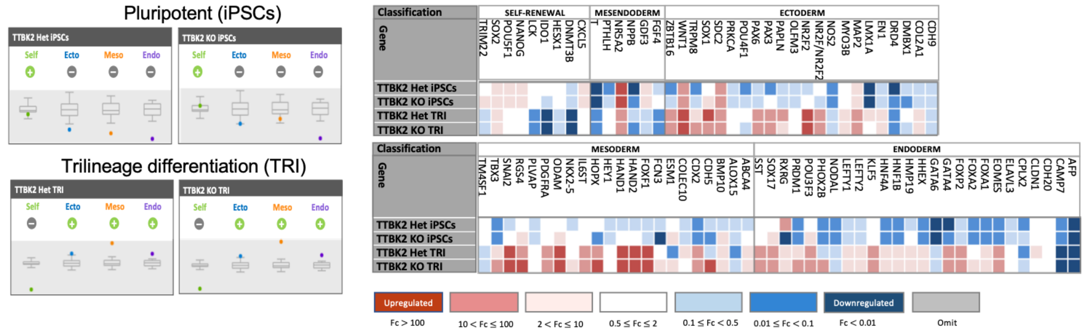

Figure S6. Generation and characterization of TTBK2 knockout iPSCs using CRISPR/Cas9.
(A) Table showing the top four sequence-based predicted off-targets of each guide RNA, which includes on-target score as a percentage, gene Ensembl ID, chromosome location, strand, genomic position on specific exons, on-target prediction (true/false), and forward and reverse primer sequences for PCR amplification. (B) Sanger sequencing of predicted off-targets shows the unmodified genomic sequences of predicted off-targets. (C) Pluripotent Taqman Scorecard analysis of homozygous (TTBK2 KO) and heterozygous (TTBK2 Het) edited cells in a pluripotent state (Pluripotent (iPSCs)) and differentiated into the three germ layers (Trilineage differentiation (TRI)). The left panel summarizes the results of gene expression for self-renewal (Self), ectoderm (Ecto), mesoderm (Meso), and endoderm (Endo) markers. The right panel shows a gene expression heatmap containing all individual markers.

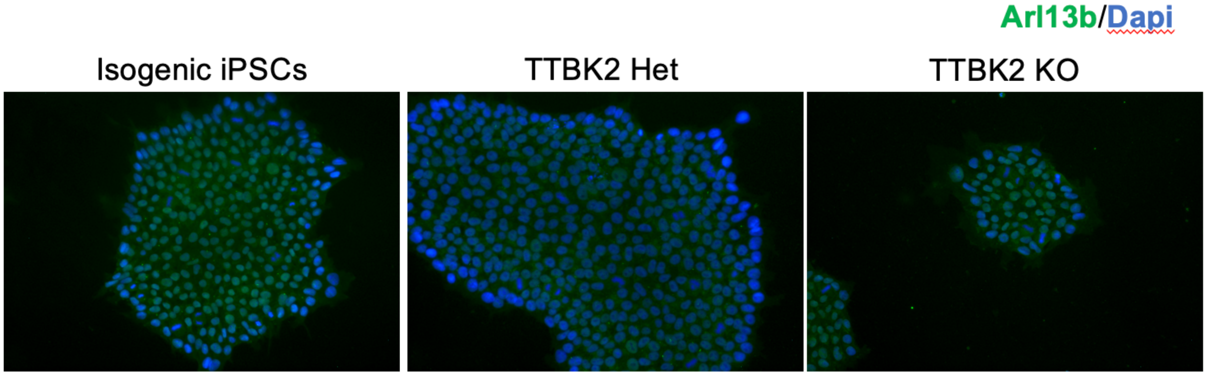

Figure S7. Primary cilia expression on TTBK2 edited clones without starvation.
Immunofluorescent staining to visualize cilia on human isogenic iPSCs, TTBK2 Het cells, and TTBK2 KO cells under standard culture conditions. A legend of the stains used to visualize cilia and nuclei is placed above the panels. Primary cilia are labeled with the Arl13B antibody (green) and the nuclei with DAPI (blue). Images were captured at 10X.

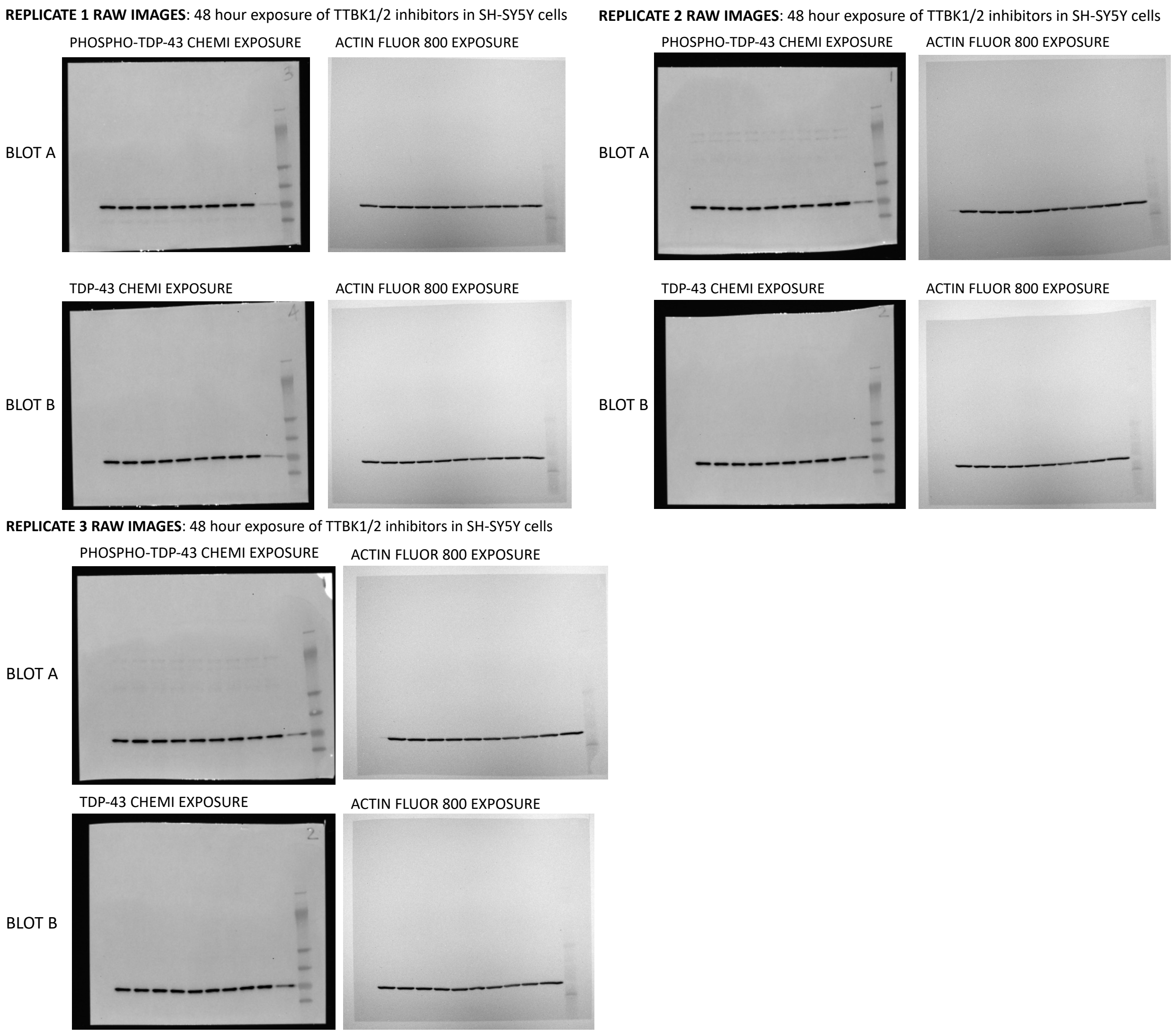

Figure S8. Unformatted western blot images corresponding to Figure 4.

RAW IMAGES FOR FIGURE S2 A&B: 24 hour exposure of TTBK1/2 inhibitors in SH-SY5Y cells

PHOSPHO-TDP-43 CHEMI EXPOSURE | ACTIN FLUOR 800 EXPOSURE

BLOT A

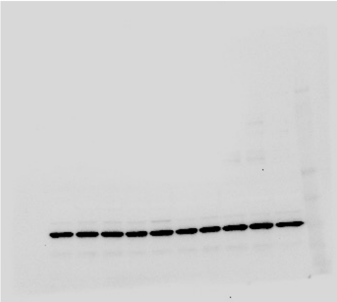

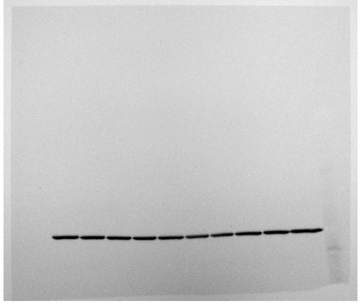

TDP-43 CHEMI EXPOSURE | ACTIN FLUOR 800 EXPOSURE

BLOT B

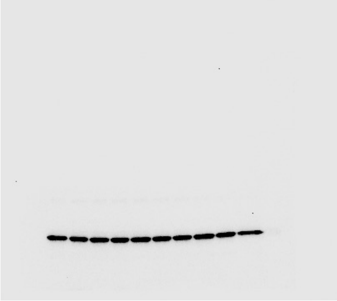

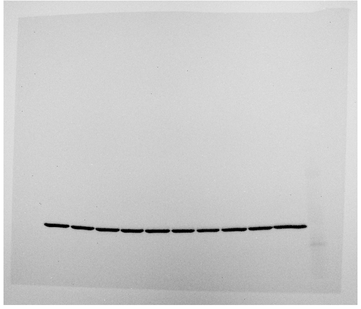

RAW IMAGES FOR FIGURE S2 C&D: 24 hour exposure of TTBK1/2 inhibitors in SH-SY5Y cells

PHOSPHO-TDP-43 CHEMI EXPOSURE | ACTIN FLUOR 800 EXPOSURE

BLOT A

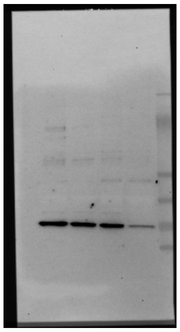

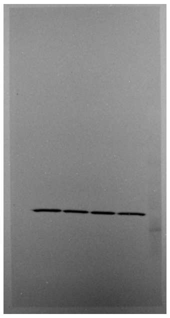

TDP-43 CHEMI EXPOSURE | ACTIN FLUOR 800 EXPOSURE

BLOT B

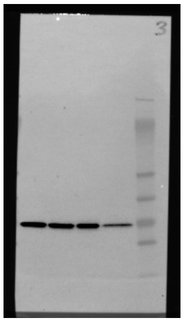

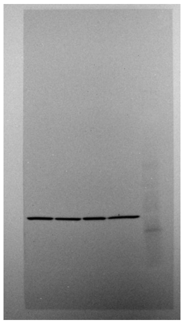

Figure S9. Unformatted western blot images corresponding to Figure S4.

RAW IMAGES: 48 hour exposure of PIKFYVE inhibitors in SH-SY5Y cells

PHOSPHO-TDP-43 CHEMI EXPOSURE

ACTIN FLUOR 800 EXPOSURE

BLOT A

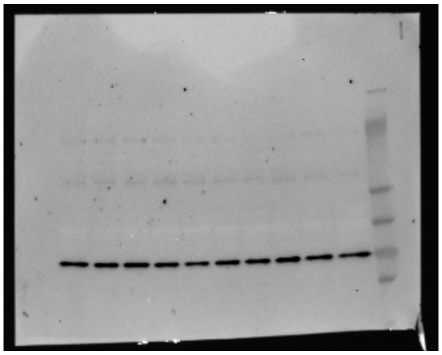

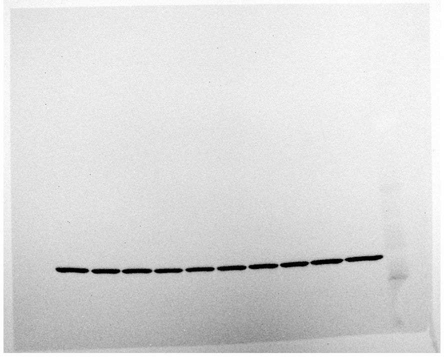

TDP-43 CHEMI EXPOSURE

ACTIN FLUOR 800 EXPOSURE

BLOT B

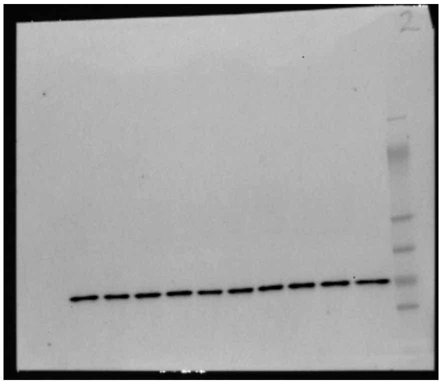

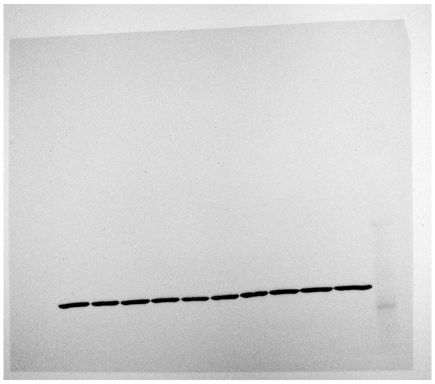

Figure S10. Unformatted western blot images corresponding to Figure S5.

Chemistry Experimental

General Information for Chemical Synthesis.

Reagents were obtained from verified suppliers and employed without purification. Temperatures are in degrees Celsius (°C); solvent was removed using a rotary evaporator; and thin layer chromatography as well as LC–MS were used to monitor reaction progress. ^{1}H NMR and additional analytical data was collected for intermediates and final compounds to verify their identity and evaluate their purity. ^{1}H and ^{13}C NMR spectra were obtained in DMSO-d_6 or MeOD-d_4 and recorded using Bruker spectrometers. Magnet strength is listed in each experimental write-up along with peak positions in parts per million (ppm). Peaks in NMR spectra are calibrated versus the shift of the indicated deuterated solvent and coupling constants (*J* values) are reported in hertz (Hz). Peak multiplicities are included as follows: singlet (s), doublet (d), doublet of doublets (dd), triplet (t), pentet (p), and multiplet (m). Purity of all compounds was assessed via HPLC.

Preparation of AMG28 as well as analogs **4**–**11** began with Fischer indole synthesis to produce **15**. Synthesis of analogs **1** and **2** was made possible from commercially available indoles **14** and **16**, respectively. Intermediates **17**–**19** were accessed from indoles **14**–**15** via selective oxidation with DDQ. Next, intermediates **20**–**22** were synthesized from **17**–**19** by refluxing with Bredereck's reagent and then cyclized to the corresponding aminopyrimidines **23**–**25** using guanidine. The desired TTBK inhibitors **1**, **2**, **4**–**11**, and AMG28 were synthesized from **23**–**25** via Sonogashira coupling conditions (Figure S3). In analogous fashion, inhibitor **3** was accessed from commerically available indole **26** using Sonogashira coupling conditions (Figure S3). Full synthetic procedures and compound characterization are included in a separate manuscript.[1] ^{1}H NMR data and spectra for the cell-active TTBK inhbitors are included below to confirm identity.

Preparation of Tracer **29** began with N-alkylation of the indole nitrogen of commerically available indole **26** with a Boc-protected propyl amine to produce **27**. Next, acidic conditions promoted the liberation of the pendant amine in intermediate **28**. A two-step sequence was employed to first install the desired alkyne bearing a propargylic alcohol via Sonogashira coupling conditions, and then attach the NanoBRET 590 dye under basic conditions and yield final tracer **29** (Figure S3). It is worth noting that the propargylic alcohol is acid labile and should not be exposed to acidic conditions, even during purification. Full characterization of the final tracer is included.

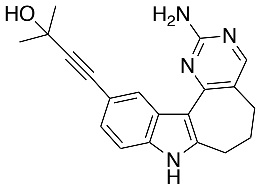

AMG28

4-(2-amino-5,6,7,8-tetrahydropyrimido[4',5':3,4]cyclohepta[1,2-b]indol-11-yl)-2-methylbut-3-yn-2-ol (AMG28). The analytical data for AMG28 matches that previously published.[1,2] ^{1}H NMR (400 MHz, DMSO-d_6) δ 11.61 (s, 1H), 8.71 (s, 1H), 8.29 (s, 1H), 7.92 (s, 1H), 7.26 (d, *J* = 8.3 Hz, 1H), 7.11 (dd, *J* = 8.4, 1.6 Hz, 1H), 6.20 (s, 2H), 3.19 – 3.15 (m, 2H), 2.66 – 2.59 (m, 2H), 1.98 – 1.92 (m, 2H), 1.50 (s, 6H).

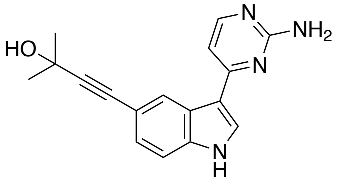

3

4-(3-(2-aminopyrimidin-4-yl)-1H-indol-5-yl)-2-methylbut-3-yn-2-ol ***(3)****.* The analytical data for **3** matches that previously published.[1,3] ^{1}H NMR (400 MHz, DMSO-d_6) δ 11.83 (s, 1H), 8.56 (s, 1H), 8.15 – 8.09 (m, 2H), 7.41 (d, *J* = 8.4 Hz, 1H), 7.17 (d, *J* = 8.9 Hz, 1H), 7.01 (d, *J* = 5.3 Hz, 1H), 6.50 (s, 2H), 1.50 (s, 6H).

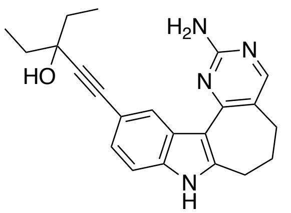

9

*1-(2-amino-5,6,7,8-tetrahydropyrimido[4',5':3,4]cyclohepta[1,2-b]indol-11-yl)-3-ethylpent-1-yn-3-ol (**9**).* The analytical data for **9** matches that previously published.[1] ^{1}H NMR (400 MHz, DMSO-d_6) δ 11.60 (s, 1H), 8.70 (d, *J* = 1.6 Hz, 1H), 7.27 (d, *J* = 8.3 Hz, 1H), 7.13 (dd, *J* = 8.2, 1.6 Hz, 1H), 6.14 (s, 2H), 5.02 (s, 1H), 3.17 (t, *J* = 6.5 Hz, 2H), 2.66 – 2.59 (m, 2H), 1.97 (dd, *J* = 10.5, 5.5 Hz, 2H), 1.75 – 1.56 (m, 4H), 1.03 (t, *J* = 7.4 Hz, 6H).

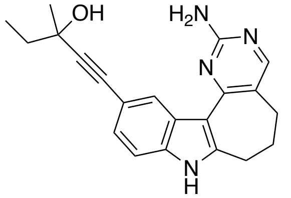

10

*1-(2-amino-5,6,7,8-tetrahydropyrimido[4',5':3,4]cyclohepta[1,2-b]indol-11-yl)-3-methylpent-1-yn-3-ol (**10**).* The analytical data for **10** matches that previously published.[1] ^{1}H NMR (400 MHz, DMSO-d_6) δ 11.60 (s, 1H), 8.71 (s, 1H), 8.16 (d, *J* = 2.2 Hz, 1H), 7.92 (s, 1H), 7.27 (d, *J* = 8.2 Hz, 1H), 7.12 (dd, *J* = 8.3, 1.7 Hz, 1H), 6.17 (s, 2H), 3.17 (t, *J* = 6.6 Hz, 2H), 2.62 (d, *J* = 9.1 Hz, 2H), 1.98 – 1.92 (m, 2H), 1.71 – 1.62 (m, 2H), 1.45 (s, 3H), 1.03 (t, *J* = 7.4 Hz, 3H).

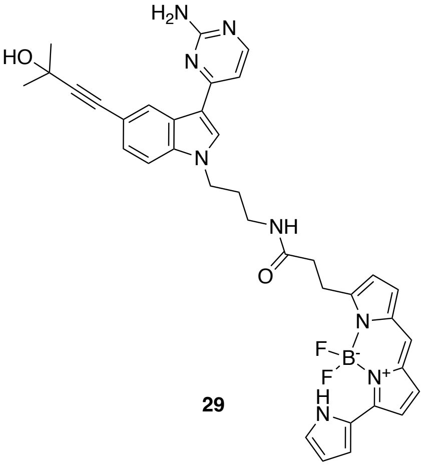

29

*N-(3-(3-(2-aminopyrimidin-4-yl)-5-(3-hydroxy-3-methylbut-1-yn-1-yl)-1H-indol-1-yl)propyl)-3-(5,5-difluoro-7-(1H-pyrrol-2-yl)-5H-5λ4,6λ4-dipyrrolo[1,2-c:2',1'-f][1,3,2]diazaborinin-3-yl)propenamide (**29**).* ^{1}H NMR (400 MHz, MeOD-d_4) δ 8.58 – 8.56 (m, 1H), 8.01 (d, *J* = 5.5 Hz, 1H), 7.99 (s), 7.37 (dd, *J* = 8.6, 0.8 Hz, 1H), 7.24 (dd, *J* = 8.5, 1.6 Hz, 1H), 7.21 – 7.17 (m, 2H), 7.14 (d, *J* = 4.6 Hz, 1H), 7.12 (s, 1H), 6.99 (d, *J* = 4.6 Hz, 1H), 6.95 (d, *J* = 5.5 Hz, 1H), 6.87 (d, *J* = 3.9 Hz, 1H), 6.36 – 6.33 (m, 2H), 4.14 (t, *J* = 7.0 Hz, 2H), 3.23 (t, *J* = 6.5 Hz, 2H), 2.67 (t, *J* = 7.5 Hz, 2H), 2.03 (p, *J* = 6.8 Hz, 2H), 1.60 (s, 6H). LCMS: calculated for $C_{36}H_{36}BF_2N_8O_2$ $[M + H]^+$: 661.30. Found: 661.34.

^{1}H Spectra for Final Compounds

^{1}H NMR (400 MHz, MeOD-d_4) 4-(2-amino-5,6,7,8-tetrahydropyrimido[4',5':3,4]cyclohepta[1,2-b]indol-11-yl)-2-methylbut-3-yn-2-ol (AMG28):

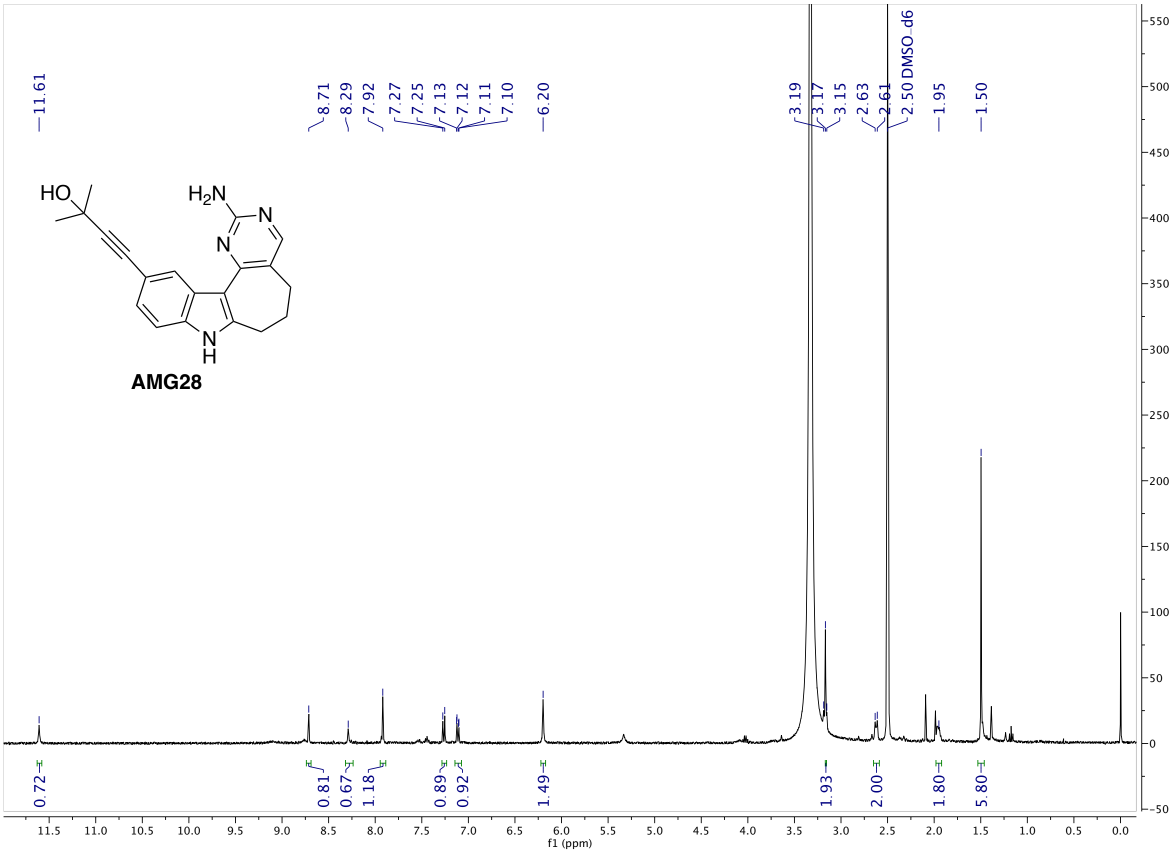

^{1}H NMR (400 MHz, MeOD-*d4*) 4-(3-(2-aminopyrimidin-4-yl)-1H-indol-5-yl)-2-methylbut-3-yn-2-ol (**3**):

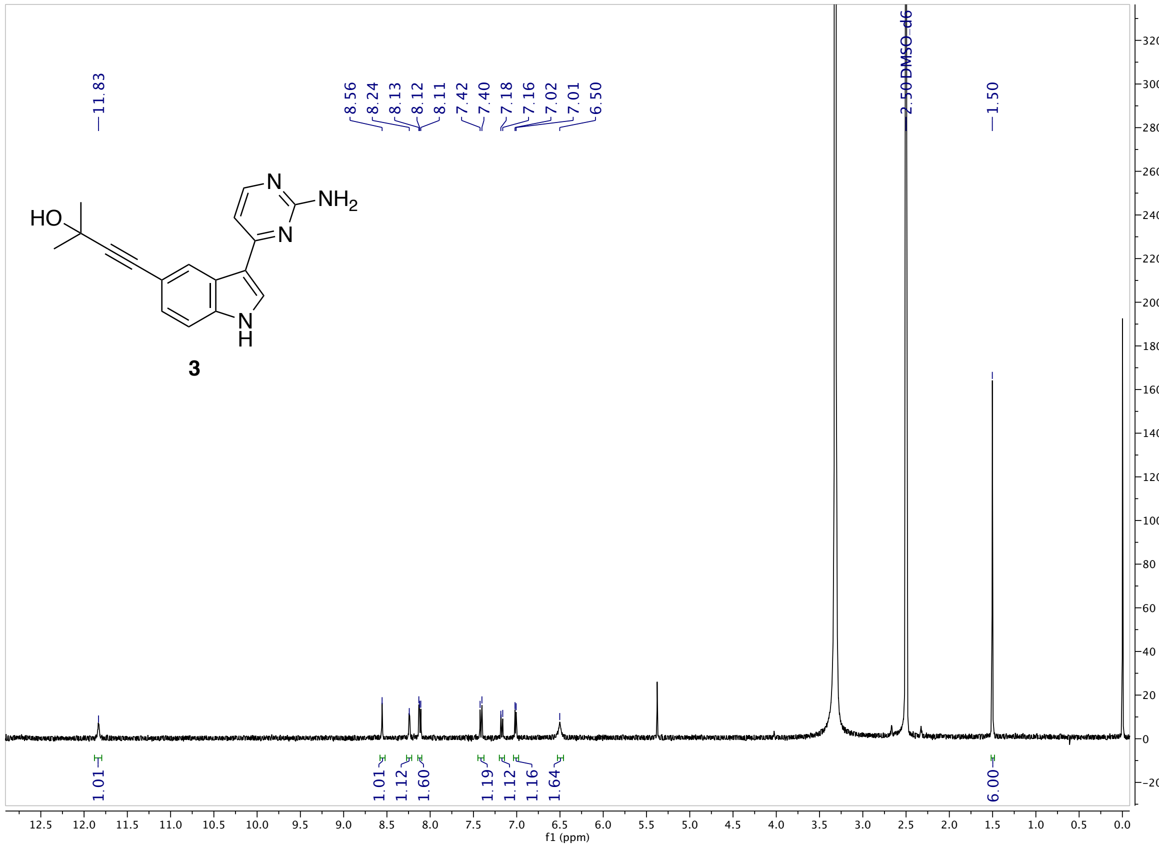

^{1}H NMR (400 MHz, DMSO-d_6) 1-(2-amino-5,6,7,8-tetrahydropyrimido[4',5':3,4]cyclohepta[1,2-b]indol-11-yl)-3-ethylpent-1-yn-3-ol (**9**):

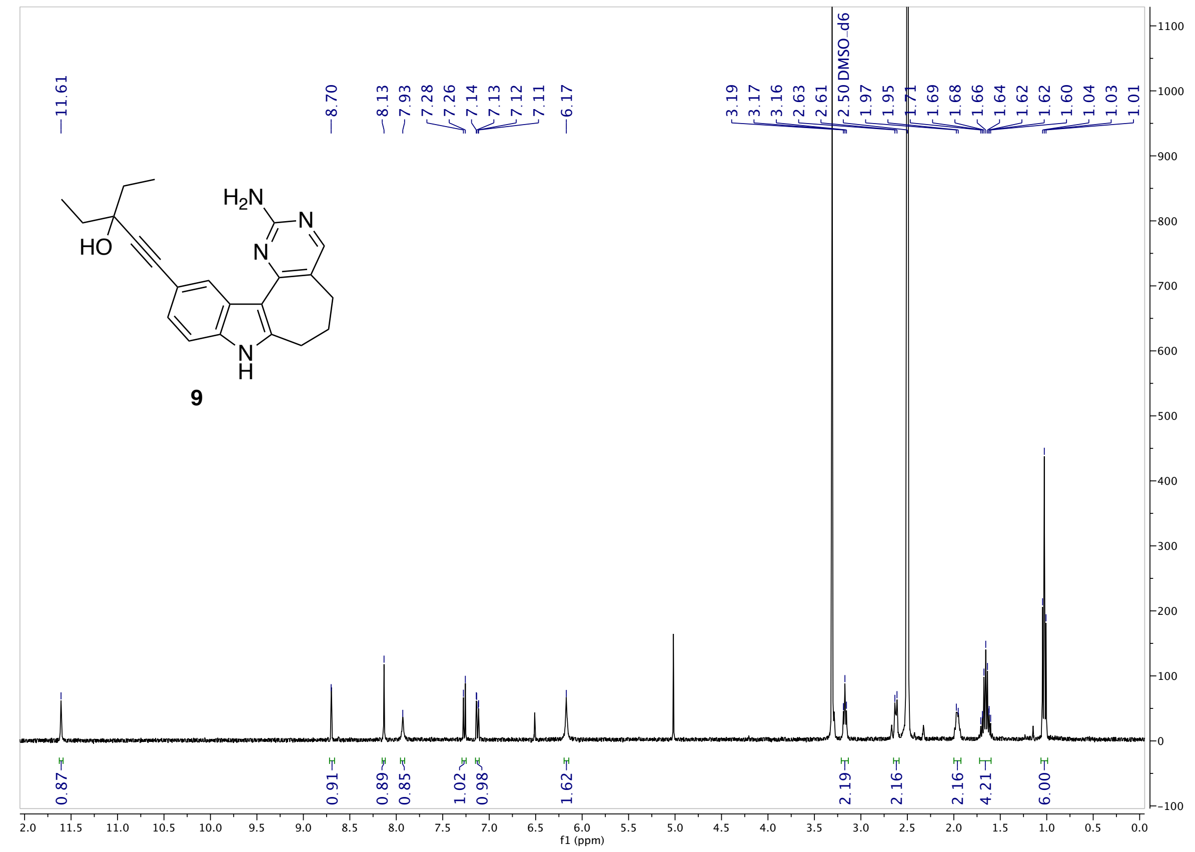

^{1}H NMR (400 MHz, MeOD-d_4) 1-(2-amino-5,6,7,8-tetrahydropyrimido[4',5':3,4]cyclohepta[1,2-b]indol-11-yl)-3-methylpent-1-yn-3-ol (**10**):

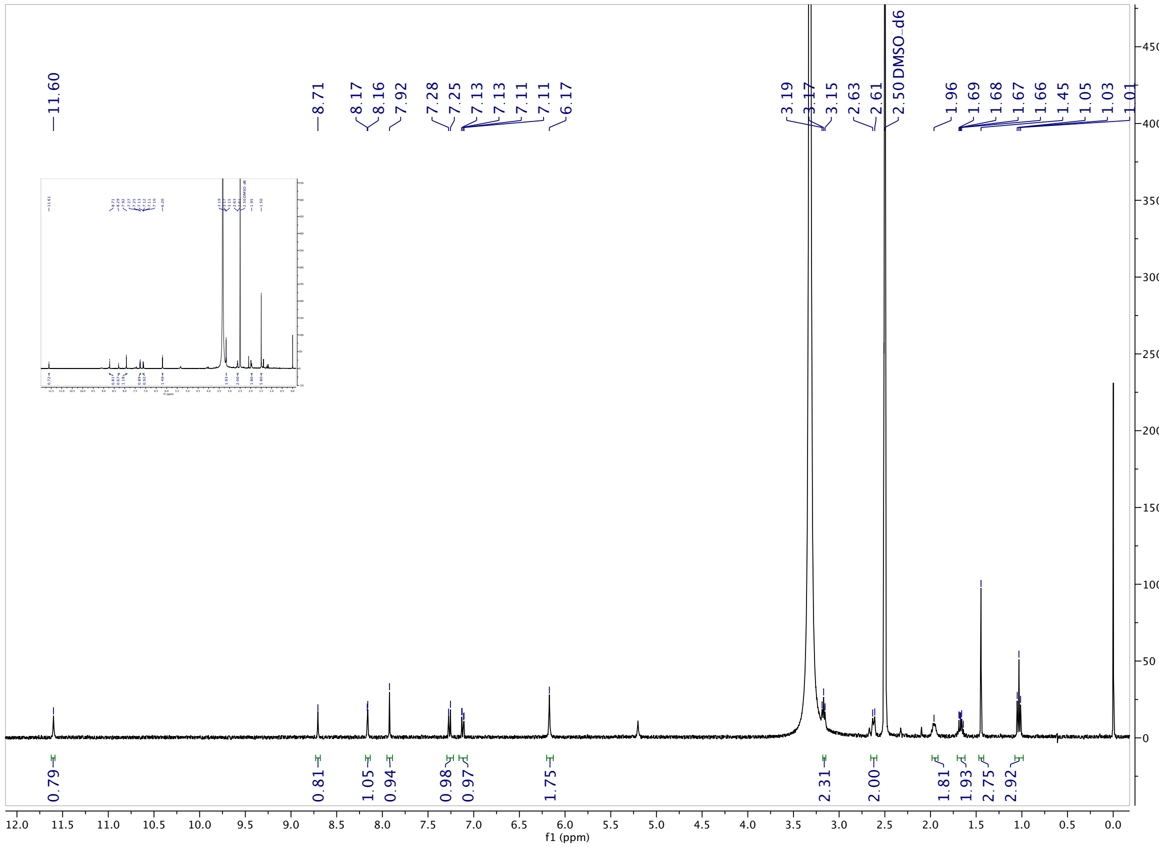

^{1}H NMR (400 MHz, MeOD-d_4) N-(3-(3-(2-aminopyrimidin-4-yl)-5-(3-hydroxy-3-methylbut-1-yn-1-yl)-1H-indol-1-yl)propyl)-3-(5,5-difluoro-7-(1H-pyrrol-2-yl)-5H-5λ4,6λ4-dipyrrolo[1,2-c:2',1'-f][1,3,2]diazaborinin-3-yl)propenamide (**29**):

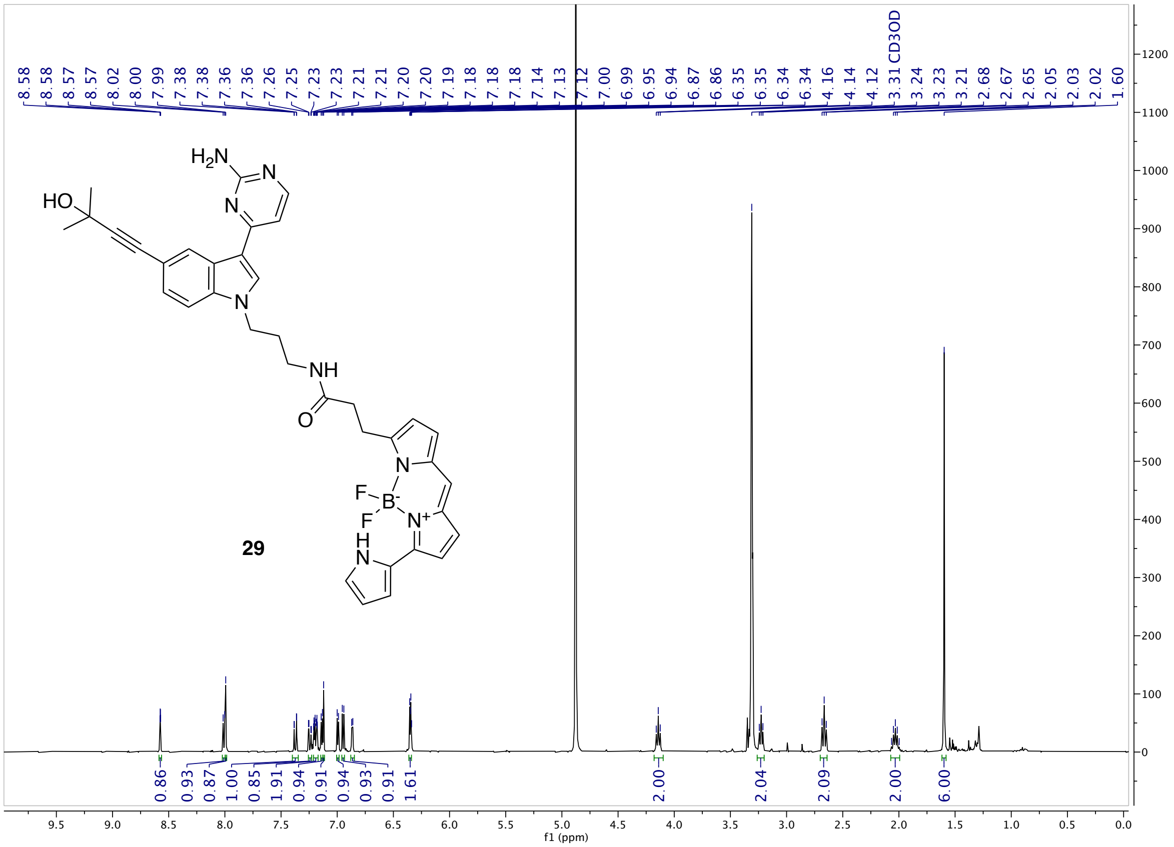

References

1 Drewry, D. H. *et al.* Identification and utilization of a chemical probe to interrogate the roles of PIKfyve in the lifecycle of β-coronaviruses. *J Med Chem*, **65**, 12860–12882. https://doi.org/10.1021/acs.jmedchem.2c00697 (2022).

2 Li, K. *et al.* Inhibiting NF-κB-inducing kinase (NIK): Discovery, structure-based design, synthesis, structure–activity relationship, and co-crystal structures. *Bioorg Med Chem Lett* **23**, 1238–1244. https://doi.org/10.1016/j.bmcl.2013.01.012 (2013).

3 Halkina, T. *et al.* Discovery of potent and brain-penetrant tau tubulin kinase 1 (TTBK1) inhibitors that lower tau phosphorylation in vivo. *J Med Chem* **64**, 6358–6380. https://doi.org/10.1021/acs.jmedchem.1c00382 (2021).
